# Zinc dynamics regulate early ovarian follicle development

**DOI:** 10.1016/j.jbc.2022.102731

**Published:** 2022-11-22

**Authors:** Yu-Ying Chen, Si Chen, Kiwon Ok, Francesca E. Duncan, Thomas V. O’Halloran, Teresa K. Woodruff

**Affiliations:** 1 Department of Obstetrics and Gynecology, Feinberg School of Medicine, Northwestern University, Chicago, Illinois, USA; 2 X-ray Science Division, Argonne National Laboratory, Lemont, Illinois, USA; 3 Department of Microbiology and Molecular Genetics, Michigan State University, East Lansing, Michigan, USA; 4 Department of Chemistry, Michigan State University, East Lansing, Michigan, USA; 5 Department of Chemistry, Northwestern University, Evanston, Illinois, USA; 6 The Chemistry of Life Processes Institute, Northwestern University, Evanston, Illinois, USA; 7 Department of Obstetrics and Gynecology, Michigan State University, East Lansing, Michigan, USA

**Keywords:** folliculogenesis, follicle, oocyte, germ cell, granulosa cell, zinc, ACN, acetonitrile, BNP, Bionanoprobe, BSA, bovine serum albumin, DPBS, Dulbecco’s phosphate buffered saline, GC, granulosa cell, GV, germinal vesicle, NSDS-PAGE, native SDS-PAGE, pfGC, primordial follicle granulosa cell, PVA, poly(vinyl)alcohol, XFM, X-ray fluorescence microscopy, ZP, zona pellucida

## Abstract

Zinc fluctuations regulate key steps in late oocyte and preimplantation embryo development; however, roles for zinc in preceding stages in early ovarian follicle development, when cooperative interactions exist between the oocyte and somatic cells, are unknown. To understand the roles of zinc during early follicle development, we applied single cell X-ray fluorescence microscopy, a radioactive zinc tracer, and a labile zinc probe to measure zinc in individual mouse oocytes and associated somatic cells within early follicles. Here, we report a significant stage-specific increase and compartmental redistribution in oocyte zinc content upon the initiation of early follicle growth. The increase in zinc correlates with the increased expression of specific zinc transporters, including two that are essential in oocyte maturation. While oocytes in follicles exhibit high tolerance to pronounced changes in zinc availability, somatic survival and proliferation are significantly more sensitive to zinc chelation or supplementation. Finally, transcriptomic, proteomic, and zinc loading analyses reveal enrichment of zinc targets in the ubiquitination pathway. Overall, these results demonstrate that distinct cell type–specific zinc regulations are required for follicle growth and indicate that physiological fluctuation in the localization and availability of this inorganic cofactor has fundamental functions in early gamete development.

The ovarian follicle, consisting of the oocyte surrounded by supporting somatic cells, progresses through well-defined developmental stages in the ovary (1). Follicle development, or folliculogenesis, begins with the activation of selective dormant primordial follicles. These primordial follicles are formed in the first few days of life in the mouse and are characterized by a nongrowing oocyte arrested at prophase of meiosis I, surrounded by one layer of mitotically arrested, squamous pre-granulosa cells (also known as primordial follicle granulosa cells; pfGCs). Through a process that is not fully understood, primordial follicle activation initiates oocyte growth, and squamous pfGCs re-enter the mitotic cycle and differentiate into cuboidal granulosa cells (GCs), forming a primary follicle with an oocyte surrounded by a single layer of GCs. As the follicle unit continues to mature, the GCs proliferate to form multiple layers and the oocyte produces an extracellular glycoprotein coat termed the zona pellucida (ZP). This is now known as a secondary follicle with specialized filopodia spanning the ZP and governing the communication between the oocyte and the GCs (2). This follicle eventually develops into a multilayer, then into a mature antral follicle with fluid filled cavity in preparation for ovulation. Upon the gonadotropin surge from the pituitary, the oocyte is released from the follicle in a process known as ovulation, and the oocyte resumes meiosis to extrude the first polar body (oocyte maturation) and again is arrested at the metaphase of meiosis II as a fertilizable mature egg (MII egg). Meiosis II is completed as a fertilized egg transitions to a zygote (3, 4). Folliculogenesis is a prerequisite for generating a high-quality gamete capable of giving rise to the next generation, yet the mechanisms governing the coordinated growth and development of the oocyte and its accompanying somatic cells during early follicle development has been elusive. This open question in development may be linked to recently uncovered roles for zinc in regulating oocyte maturation, fertilization, and early preimplantation embryo development (5, 6, 7).

Total zinc concentration corresponds to the sum of bound zinc, that is, zinc ions that are tightly bound in protein active sites, and free zinc, that is, pools of weakly bound, labile zinc complexes which are readily detected using fluorescent probes. The best understood pools of labile zinc have been described in secretory compartments (8). Like calcium, labile zinc is typically maintained at exceedingly low concentrations in the cytosol of most cells in a resting or unperturbed state, at pM to nM range (9, 10). This baseline feature allows temporal fluxes in ion release from an intracellular zinc store or from the extracellular space to diffuse and be loaded onto target proteins in signaling pathways, initiating “zinc signaling” (9, 10, 11). We and others have demonstrated that such transient fluctuations in zinc regulate several important developmental transitions in vertebrate and invertebrate oocytes, eggs, and preimplantation embryos. The cyclic change in zinc content or availability differs between species, but a murine oocyte increases its total zinc content by 20 billion atoms (*i.e.*, a 50% increase) as it completes meiosis I and then ejects ca 20% before it can complete meiosis II. Total zinc atom number peaks at metaphase II in mouse (5): this influx establishes the second meiotic arrest when EMI2 protein, a meiosis inhibitor that acquires two zinc ions and adopts a conformation that inhibits the activity of the anaphase-promoting complex/cyclosome (5, 12, 13, 14). Upon fertilization, 10 billion zinc atoms are rapidly exocytosed from zinc-loaded vesicles into the extracellular space. These ‘zinc sparks’ expose the egg ZP to ca. 10^−4^ M zinc ions result in structural changes that likely contribute to the block to polyspermy (6, 8, 15). The lowering of intracellular zinc initiates re-entry into the meiotic cell cycle and is a conserved hallmark of fertilization among different mammalian species including human (6, 16, 17). Meanwhile, zinc homeostasis is also required for the first mitotic divisions of the preimplantation embryo (7). These discoveries demonstrate that zinc is a central developmental switch. However, it is unknown if zinc similarly plays a role in early ovarian follicle development, specifically when the follicle transitions from nongrowing to rapidly growing phases.

To examine possible roles for zinc in early follicle development, we first determined zinc content and distribution in murine follicles of primordial to secondary stages. Changes in total and labile zinc pools were evaluated using quantitative single-cell imaging for intact follicles, isolated oocytes, and individual somatic cells. Single cell X-ray fluorescence microscopy (XFM) provides quantitative maps for total populations of several essential elements, while a radioactive zinc tracer method and confocal imaging with a zinc-specific probe ZincBY-1 (8) allow interrogation of dynamic changes in labile zinc content. RNA *in situ* hybridization and immunofluorescence staining were used to evaluate changes in zinc transporter expression in both germ and somatic cells across this developmental sequence. We established that zinc is the most abundant transition metal in early follicles, and it increases during follicle development *via* active accrual. On a single-cell basis, total zinc increases in the oocyte during follicle development in a follicle-stage but not cell size–dependent manner, and labile zinc redistributes in specific cellular compartments. Within the oocyte, the expression of several zinc transporters increases during follicle development, whereas total zinc levels and transporter expression remain constant in individual somatic cells. Limiting zinc during early follicle development resulted in somatic cell apoptosis, which in turn decreased secondary follicle number but had little direct effect on oocyte viability. Conversely, zinc supplementation activated growth pathways in the somatic cell and increased somatic proliferation. Potential zinc partner proteins in the oocyte, identified *via* a combination of *in silico* transcriptomic analysis and autoradiography and proteomic methods, include an enrichment of targets in the ubiquitination pathways. These results demonstrate that zinc undergoes dynamic changes during follicular activation and growth, the oocyte and somatic cells of the follicles exhibit distinct zinc regulatory mechanisms, and zinc homeostasis is required for normal folliculogenesis.

## Results

### The total zinc content increases during follicle development *via* active accrual

**Figure 1:**
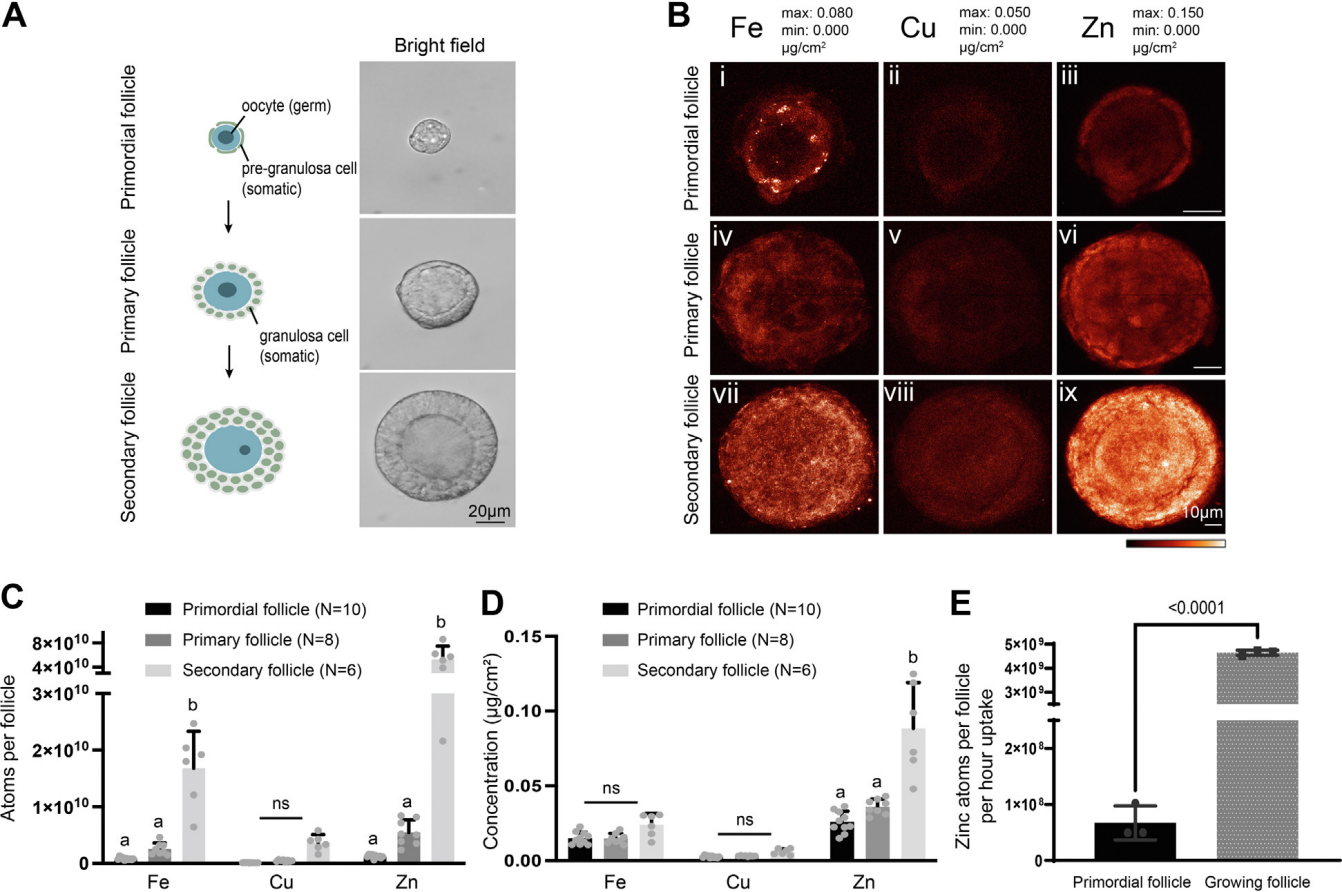
**Total zinc content increases during follicle development *via* active accrual.***A*, illustration and bright field images of ovarian primordial, primary, and secondary stage follicles. The scale bar represents 20 μm. *B*, elemental maps of iron, copper, and zinc for each follicular stage established using synchrotron-based X-ray fluorescence microscopy. The color scale bar represents the minimum and maximum elemental contents (μg/cm^2^) of each element. The scale bar represents 10 μm. *C*, quantification of total atom number of iron, copper, and zinc from the elemental maps, with whole follicle area as the region of interest (ROI). *D*, quantification of elemental concentration in μg/cm^2^ of iron, copper, and zinc from the elemental maps. *E*, total zinc atom per follicle per hour uptake in the primordial and growing follicles of primary and secondary follicle stages measured using a Zn-65 uptake method. Data represent mean values (SD). N= 10 primordial follicles, 8 primary follicles, 6 secondary follicles (*B–D*), and 3 independent repeats of Zn-65 uptake experiments on pools of 100 to 500 primordial follicles and 200 growing follicles (*E*). Letters denote statistically significant differences between developmental stages for each element by two-way ANOVA with Tukey’s multiple comparison test (*C* and *D*, *p* < 0.05) (*C*: Secondary to primary stage total iron P_adj_ = 0.0004, total zinc P_adj_ < 0.0001) (*D*: Secondary to primary stage zinc concentration P_adj_ < 0.0001) or unpaired *t* test (*E*). See Table S1 for corresponding quantification.

To characterize potential roles of zinc during early ovarian folliculogenesis, we first evaluated total zinc content in individual primordial, primary, and secondary follicles (Fig. 1*A*) using synchrotron-based XFM at the Bionanoprobe (BNP) of Argonne National Laboratory (18). XFM at the BNP provided the total elemental content and distribution in follicles through detection of the X-ray fluorescence spectra that were characteristic of each element, which were then calibrated and presented in 2D images with the concentration unit of μg/cm^2^ or converted to the number of atoms per region of interest (Fig. 1*B*). Using this technology, we determined that zinc, along with two other major trace elements iron and copper, were found in all follicle stages, with zinc and iron being more abundant than copper (Fig. 1*B*). Following quantification, we measured total atom number within the follicle throughout folliculogenesis, with highest metal content observed in secondary follicles (Table S1). Specifically, zinc was more abundant than iron and copper in follicles in each of the stages examined, and the total number of zinc atoms per follicle increased across the primordial, primary, and secondary stages (Fig. 1*C*). Because the follicle expands in size and cell number during follicle development, we normalized total atom number to whole follicle area (Fig. S1*A*) and calculated the elemental concentration in μg/cm^2^. This analysis demonstrated that the greatest magnitude of change among the transition metals occurred in zinc during folliculogenesis. The concentration of zinc increased significantly (3.4-fold) from the primordial to secondary stage; iron and copper concentrations increased to a lesser extent (1.6-fold and 2.2-fold, respectively) (Fig. 1*D*).

To determine whether the increase in zinc content observed in growing follicles (primary and secondary follicles with one or two layers of GCs) was due to changes in active zinc uptake during follicular development, we compared uptake of the radiotracer Zn-65 in live follicles between primordial and growing follicles, as opposed to XFM measurement performed on ‘fixed’ samples. The total zinc uptake was 6.7 × 10^7^ and 4.6 × 10^9^ atoms/h normalized to a single primordial and a single growing follicle, respectively (Figs. 1*E* and S2). Thus, growing follicles rapidly acquired zinc at rates approximately 70 times greater than primordial follicles, suggesting a potentially important role of zinc during early follicle development.

### Total zinc content increases in the oocyte during folliculogenesis but not in individual somatic cells

**Figure 2:**
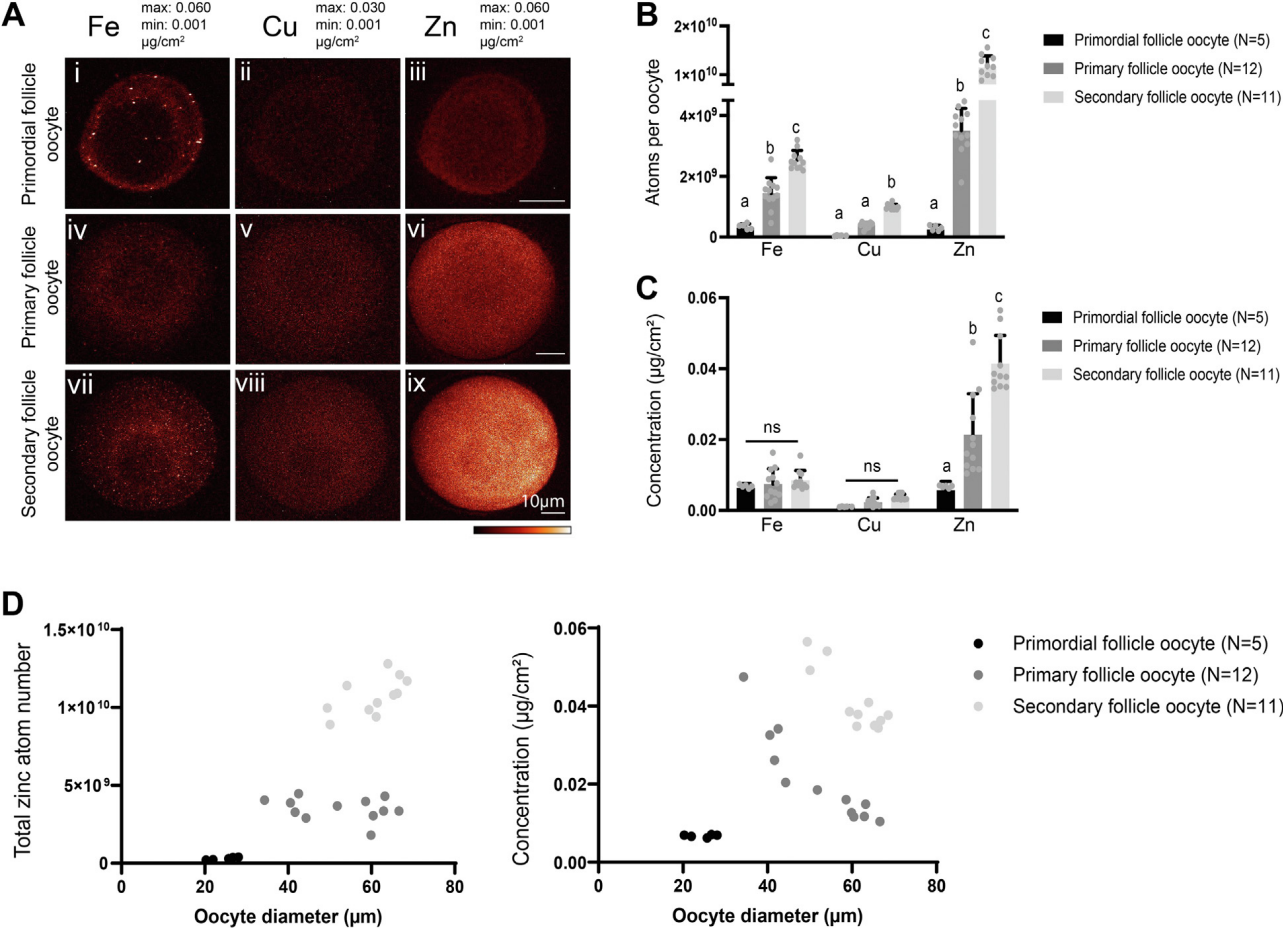
**Total zinc content increases in the oocyte in a follicle stage-specific manner.***A*, elemental maps of iron, copper, and zinc for single oocytes isolated from primordial, primary, and secondary stage follicles and analyzed by synchrotron-based X-ray fluorescence microscopy. The color scale bar represents the minimum and maximum elemental contents (μg/cm^2^) of each element. The scale bar represents 10 μm. *B*, quantification of total atom number of iron, copper, and zinc from the elemental maps, with whole oocyte area as the ROI. *C*, quantification of elemental concentration in μg/cm^2^ of iron, copper, and zinc in the oocyte from the elemental maps. *D*, plots of total zinc atom number (*left*) and zinc concentration (*right*) in the oocytes of all three follicle stages against the diameter of the oocytes. Data represent mean values (SD). N = 5 primordial follicle oocytes, 12 primary follicle oocytes, 11 secondary follicle oocytes (*A–D*). Letters denote statistically significant differences between developmental stages of each element by two-way ANOVA with Tukey’s multiple comparison test (*B* and *C*, *p* < 0.05) (*B*: Primary to primordial stage total iron P_adj_ = 0.0017, total zinc P_adj_ < 0.0001; Secondary to primary stage total iron P_adj_ <0.0001, copper P_adj_ = 0.0363, zinc P_adj_ < 0.0001) (*C*: Primary to primordial stage and secondary to primary stage zinc concentration P_adj_ < 0.0001). See Table S2 for corresponding quantification. ROI, region of interest.

To characterize metal distribution in the oocytes and the somatic cells within a follicle separately, we dissociated individual oocytes and somatic cells for XFM analysis by enzyme digestion after follicle isolation (19). The metal content of iron, copper, and zinc within the oocyte all increased significantly during follicle development (Fig. 2*A* and Table S2). At the primordial stage, the oocyte zinc atom number was higher than copper but was comparable to the iron level initially, and zinc did not become the most abundant transition metal until the primary follicle stage and beyond. Specifically, during the primordial to primary stage transition, zinc showed the greatest increase than other metals or stages, an increase of 14-fold (Fig. 2*B*). Upon analyzing metal concentrations, iron and copper remain at a similar level from primordial to secondary follicle stages, suggesting that the oocyte accrued these metals at a similar rate as it grew in size across stages (Fig. 2*C*). On the contrary, zinc concentration significantly increased in the oocyte throughout follicle development, with zinc levels 6- to 11-fold more concentrated than copper in the three stages (Fig. 2*C*). As the oocyte differs in size even within the same follicle stage, we further analyzed the relationship between zinc level and oocyte size based on diameter. Interestingly, the increase of zinc in the oocyte is not a simple linear function of oocyte size. Pearson correlation coefficients were 0.92, −0.29, and 0.60 for oocyte diameter *versus* zinc content in the primordial, primary, and secondary follicle stages, respectively. This shows that the increase in zinc in the oocyte seemed to track with developmental stage rather than a continuous increase with cell size (Fig. 2*D*). This can be seen clearly for oocytes in the 30 μm size range where oocytes within primary follicles have a 10-fold higher zinc content than those in primordial follicles. Furthermore, oocytes within secondary follicles in the 60 μm size range have 3-fold more zinc than those in primary follicles of the same size (Fig. 2*D*).

**Figure 3:**
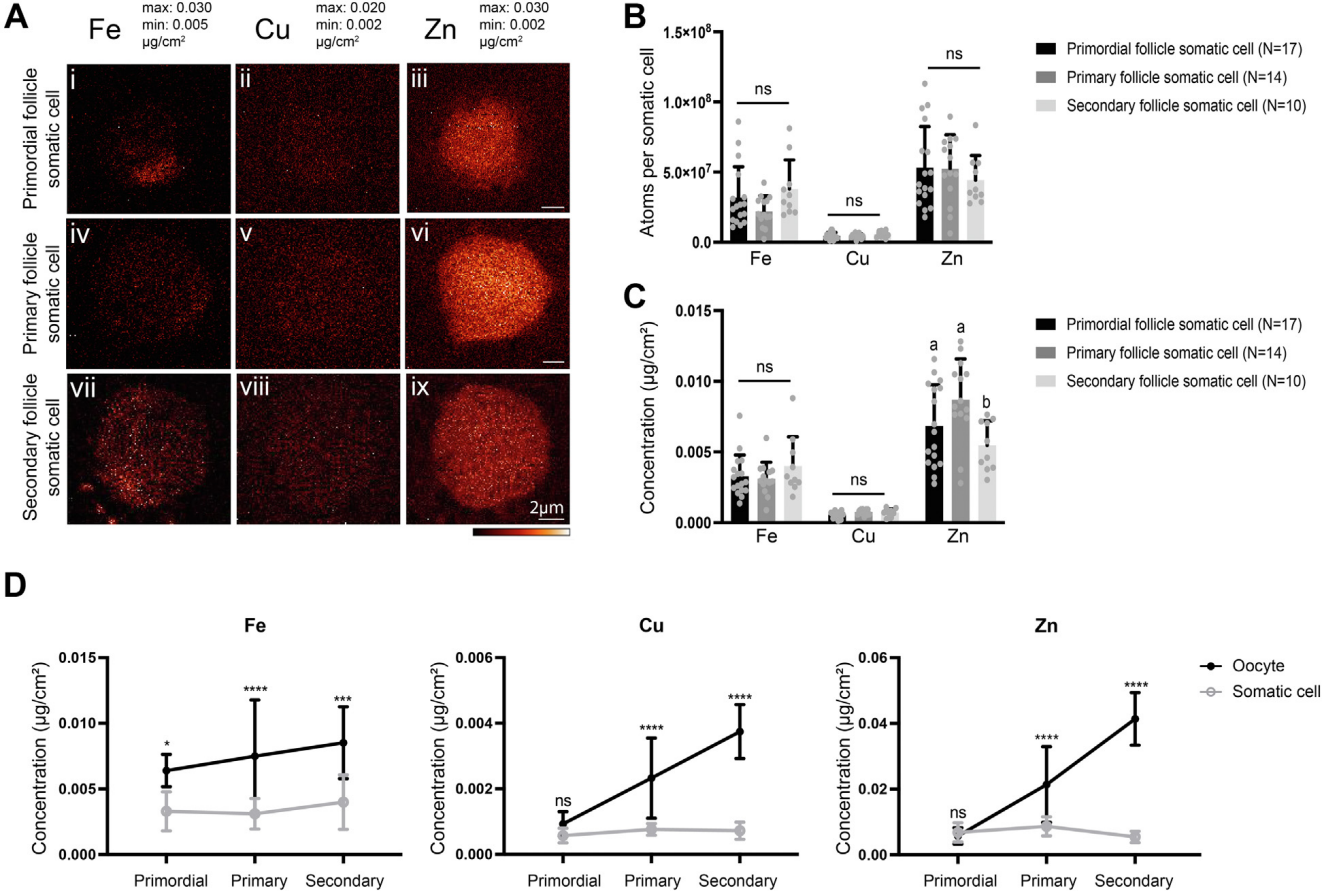
**Total zinc content does not increase in individual somatic cells during follicle development.***A*, elemental maps of iron, copper, and zinc for single somatic cells isolated from primordial, primary, and secondary stage follicles and analyzed by synchrotron-based x-ray fluorescence microscopy. The color scale bar represents the minimum and maximum elemental contents (μg/cm^2^) of each element. The scale bar represents 2 μm. *B*, quantification of total atom number of iron, copper, and zinc from the elemental maps, with whole somatic cell area as the ROI. *C*, quantification of elemental concentration in μg/cm^2^ of iron, copper, and zinc in the somatic cells from the elemental maps. *D*, comparison of iron, copper, and zinc concentrations in single oocytes *versus* individual somatic cells from the three follicle stages. Data represent mean values (SD). N = 17 pfGCs, 14 primary follicle GCs, 10 secondary follicle GCs (*A–C*). Letters denote statistically significant differences between developmental stages of each element by two-way ANOVA with Tukey’s multiple comparison test (*B* and *C*, *p* < 0.05) (*C*: Secondary to primary stage zinc concentration P_adj_ < 0.0001) or Śídák’s multiple comparisons test comparing between the two cell types (*D*, ∗*p*= 0.0152, ∗∗∗*p*= 0.0002, ∗∗∗∗*p*< 0.0001). See Table S3 for corresponding quantification. GC, granulosa cell; pfGC, primordial follicle granulosa cell; ROI, region of interest.

In contrast to the oocyte, the elemental content in single somatic cells did not increase during follicle development (Fig. 3, *A* and *B* and Table S3). After being dissociated from the oocyte, the squamous pfGCs from primordial follicles rounded up and were similar in size to the GCs in primary and secondary follicles (Fig. S1*C*), and the elemental content did not change during differentiation from pfGCs to GCs (Fig. 3*B*). Moreover, upon analyzing metal concentrations, zinc concentration decreased in the somatic cells of secondary follicles, while iron and copper concentration remained at a similar level during follicle development (Fig. 3*C*). To better understand metal dynamics in the germ cell and the somatic cell directly, we compared iron, copper, and zinc concentrations in single oocytes *versus* single somatic cells across follicle development. While the metal concentrations in the somatic cells stayed constant, those in the oocyte increased significantly. Significant differences were observed between somatic cells and the oocytes in the primary follicle. By the secondary follicle stage, the iron concentration in the oocyte was twice the amount of a somatic cell, the copper concentration was 5-times higher despite being an order magnitude lower than zinc, and the zinc concentration was 7.6-times higher in the oocyte (Fig. 3*D*). Finally, we overlaid and compared the total zinc content of the oocyte in whole follicles. We observed that around 20% of zinc in a primordial follicle was in the oocyte compartment. This number increased to about 60% in the primary stage and decreased to 20% in a secondary follicle (Fig. S3*A*). In a secondary follicle, even though the zinc content did not change in individual GCs, there are many more somatic cells during this stage; therefore, the main zinc increase was observed in the somatic compartment as a whole (Fig. S3*B*).

Overall, these results indicate that the changes in zinc content during follicle development are much more extensive in the oocyte relative to individual somatic cells. Moreover, the oocyte-specific increase in zinc appears to be follicle class–dependent and not just a simple consequence of the increase in oocyte size that occurs during oogenesis. Lastly, even though somatic zinc content did not change on an individual cell basis, most of the zinc was allocated to the somatic compartment as GC number increases in the secondary follicle.

### Labile zinc undergoes subcellular reorganization in the oocyte during follicle development

**Figure 4:**
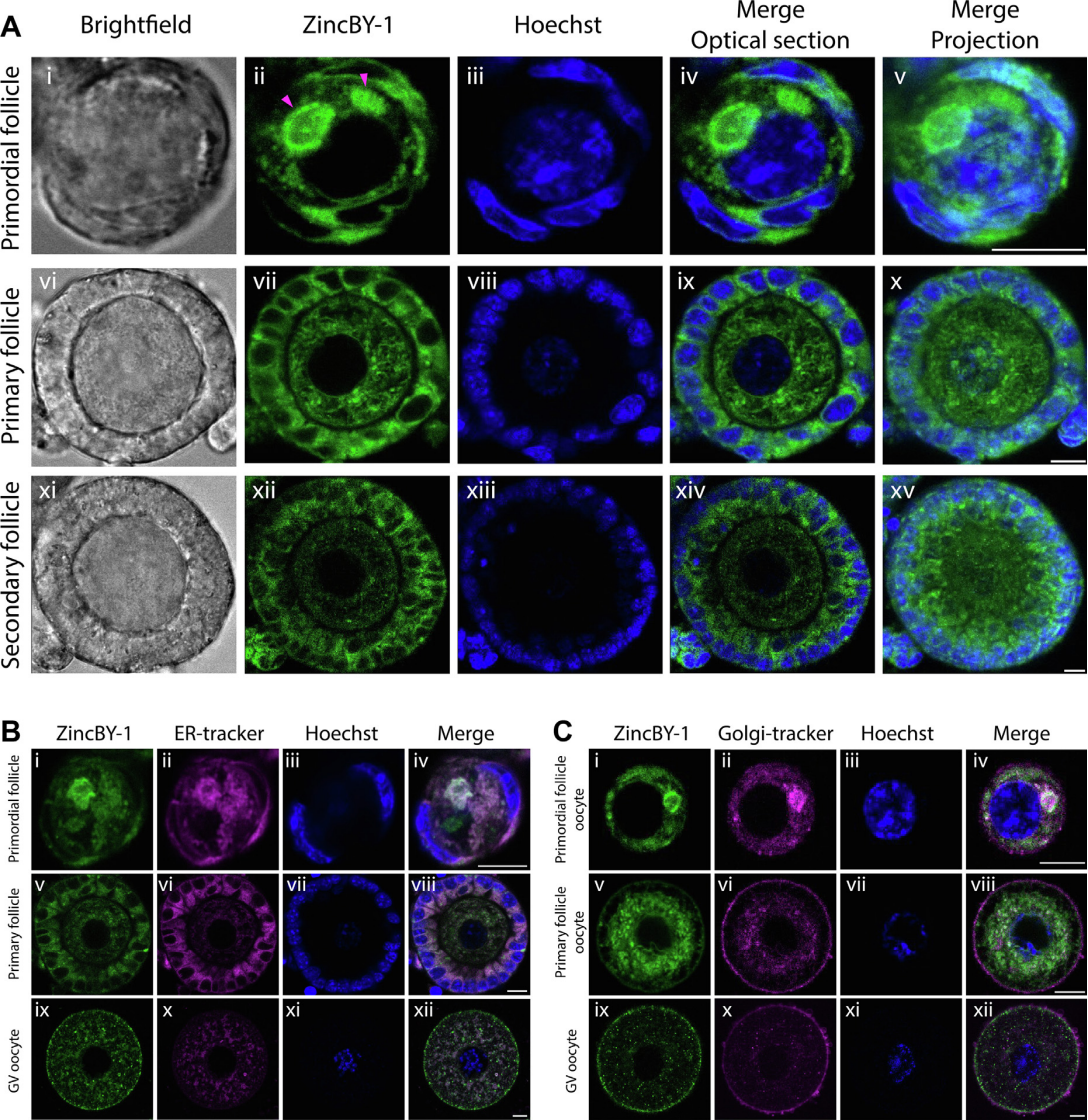
**Labile zinc in the oocyte undergoes subcellular reorganization during follicle development.***A*, representative images of labile zinc staining using ZincBY-1 (*green*) and Hoechst DNA stain (*blue*) on live primordial follicles (i–v), primary follicles (vi–x), and secondary follicles (xi–xv) as shown in brightfield (i, vi, xi), confocal optical slices of individual or merged channels (ii–iv, vii–ix, xii–xiv), and Z-stack projection (v, x, xv). The localized cytoplasmic staining pattern was marked by *magenta arrows* (ii). *B*, confocal optical sections of live ZincBY-1 (*green*) staining colabeling with ER-tracker (*magenta*) and Hoechst (*blue*) in primordial follicles (i–iv), primary follicles (v–viii), and GV oocytes (ix–xii). *C*, confocal optical sections of live ZincBY-1 (*green*) staining colabeling with Golgi-tracker (*magenta*) and Hoechst (*blue*) in oocytes from primordial follicles (i–iv), primary follicles (v–viii), and GV stage (ix–xii) as shown in individual or merged channels. The scale bar represents 10 μm (*A–C*). See Table S4 for quantification. GV, germinal vesicle.

Intracellular zinc can be classified into two populations: protein-bound zinc and labile zinc which contributes to signaling events. Labile zinc can be readily visualized using intracellular zinc probes such as ZincBY-1, which is composed of a BODIPY core and a polypyridine zinc chelator (8). To complement our XFM studies that examined the total zinc pool (both bound and labile portion), we analyzed labile zinc populations directly by ZincBY-1 staining and confocal imaging on live follicles from the primordial to secondary stages. Labile zinc was identified in all stages of follicle development (Fig. 4*A*). Within primordial follicles, highly localized cytoplasmic structures enriched in labile zinc were observed in the oocytes (Fig. 4*A*, ii, marked with magenta arrows). This localized pattern was more diffuse in the oocytes of primary and secondary follicles (Fig. 4*A*, vii, xii). In contrast, the pattern of labile zinc signals in the cytoplasm of the somatic cells was similar across all stages.

The enrichment of labile zinc within perinuclear structures of the oocyte in the primordial follicle led us to examine compartmentalization possibilities. A distinguishing feature of the oocyte in a primordial follicle is the extensive colocalization of organelles such as endoplasmic reticulum (ER), Golgi, mitochondria, and mRNAs, forming a condensed structure called the Balbiani body (B-body) (20). Following follicle activation, the B-body deforms, and the organelles become evenly distributed in the cytoplasm of an oocyte (21). In mammalian cells, labile zinc is reported to be stored in ER, Golgi apparatus, mitochondria, or zinc vesicles. To determine whether the labile zinc pattern colocalizes with a subset of cellular organelles, we costained follicles with ZincBY-1 and ER-tracker, Golgi-tracker, and mitotracker. In primordial follicles, labile zinc colocalized with ER in both the oocyte and the somatic cells (Fig. 4*B*, iv), and with Golgi in the oocyte (Fig. 4*C*, iv), but not with mitochondria (Fig. S4, See Table S4 for Pearson correlation coefficient). The colocalization of labile zinc with ER continued in both the oocyte and the somatic cells in the primary follicle (Fig. 4*B*, viii), but the correlation decreased significantly in a germinal vesicle (GV) oocyte (oocytes collected from multilayer to antral follicle stage, Fig. 4*B*, xii), suggesting that labile zinc in the oocyte toward later stages distributed outside of ER. The observation was similar for labile zinc in the oocyte with Golgi-tracker staining (Fig. 4*C*, viii, xii). Overall, cell-type–specific distributions in both total and labile zinc pools are dynamic during follicle development. Specifically, we characterized ER and Golgi as potential primary labile zinc stores in early-stage oocytes but not in late-stage oocytes.

### Identification of oocyte- and somatic-specific subcellular zinc transporter expression

**Figure 5:**
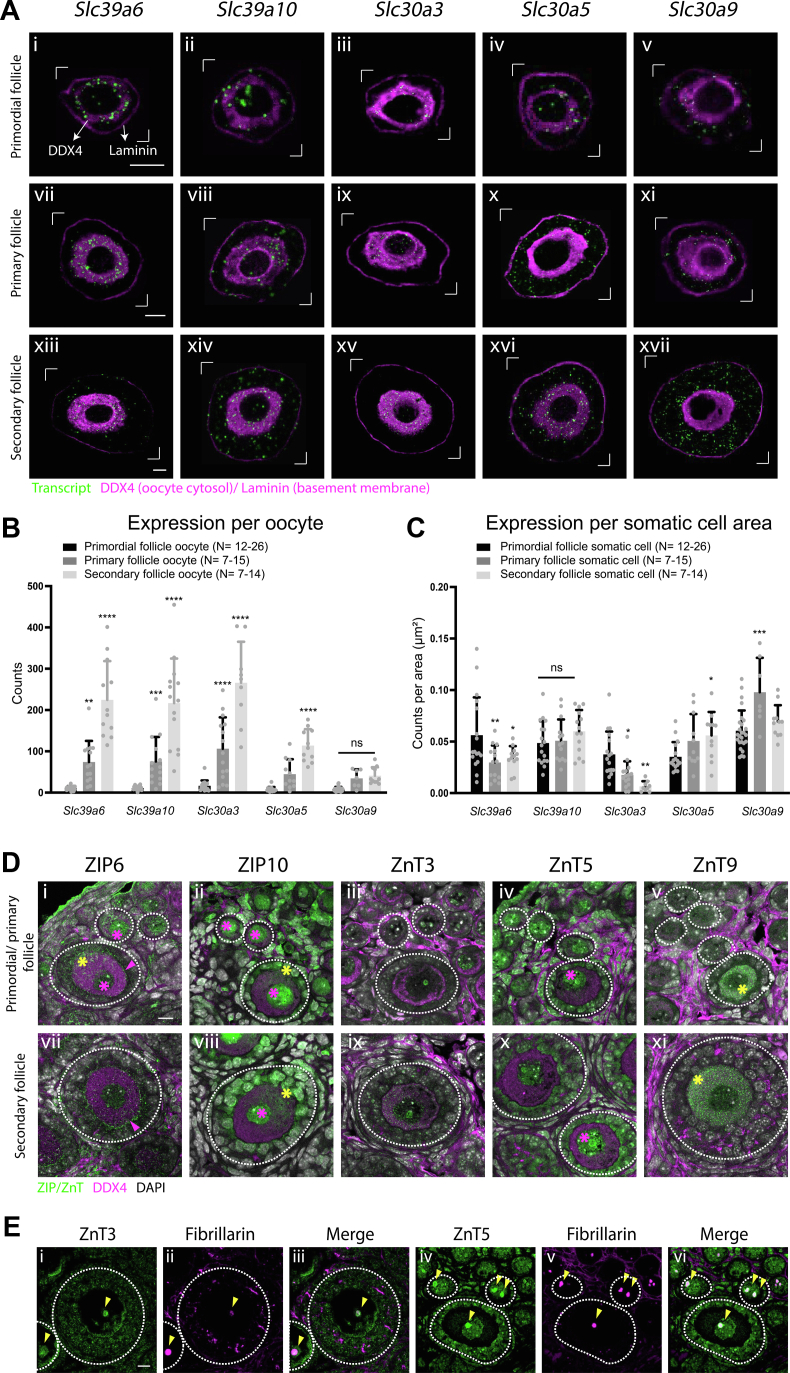
**Identification of oocyte- and somatic cell-specific subcellular zinc transporter expressions.***A*, confocal images of zinc importer genes (*Slc39a6*, *Slc39a10*) and exporter genes (*Slc30a3*, *Slc30a5*, *Slc30a9*) expression analyzed by fluorescence *in situ* hybridization combined with immunofluorescence staining on ovarian sections. The *green* puncta represent the amplification of the transcripts, while *magenta* is the combined staining of DDX4 (oocyte) and Laminin (basement membrane), which marks the boundary of the oocyte *versus* the somatic cells. Data are contained within the *white brackets*. The scale bar represents 10 μm. *B*, quantification of the puncta by counts in the oocyte of each follicle stage. *C*, quantification of the puncta in the somatic cells of each follicle stage normalized to somatic cell area (space in between oocyte and the basement membrane). *D*, confocal images of zinc importers (ZIP6, ZIP10) and exporters (ZnT3, ZnT5, ZnT9) expression analyzed by immunofluorescence staining on ovarian sections. The transporters are shown in *green*, DDX4, the costained oocyte marker is shown in *magenta*, with DNA shown in *gray*. The *yellow asterisks* mark a cytoplasmic staining pattern, and the *magenta asterisks* highlight the nuclear staining. The *magenta arrows* mark the plasma membrane of the oocyte. The scale bar represents 10 μm. *E*, confocal images of ZnT3 and ZnT5 expression colabeled with a nucleolar marker, fibrillarin, in primary and secondary follicles analyzed by immunofluorescence staining. ZnT3 and ZnT5 are shown in *green*, and nucleolar marker is shown in *magenta*. *Yellow arrows* mark the nucleolar staining patterns. The scale bar represents 10 μm. Data represent mean values (SD). N = 7 to 26 oocytes or somatic cells of each follicle stage. Statistically significant differences between growing and primordial stages of each transporter gene were computed by two-way ANOVA with Tukey’s multiple comparison test (∗*p*< 0.05, ∗∗*p*< 0.01, ∗∗∗*p* < 0.001, ∗∗∗∗*p*< 0.0001) (*B*: Primary to primordial stage *Slc39a6* P_adj_ = 0.0013, *Slc39a10* P_adj_ = 0.0010) (*C*: Primary to primordial stage *Slc39a6* P_adj_ = 0.0022, *Slc30a3* P_adj_ = 0.0251, *Slc30a9* P_adj_ = 0.0002; Secondary to primordial stage *Slc39a6* P_adj_ = 0.0201, *Slc30a3* P_adj_ = 0.0016, *Slc30a5* P_adj_ = 0.0375). *White dashed circles* mark the boundary of individual follicles (*D* and *E*).

To understand the cellular machinery involved in maintaining intracellular zinc level, we analyzed the expression pattern of zinc transporters and zinc-responsive metallothionein genes during follicle development. Over 24 solute-specific transporters involved in zinc compartmentalization are encoded in the mouse and human genome. Primary zinc transporter proteins in mammals such as the Zrt, Irt-like protein/solute carrier family 39 (ZIP/SLC39A) increases cytosolic zinc concentration by importing zinc from the extracellular space into the cell or from a zinc store to the cytosol. In contrast, zinc transporter/SLC30A family proteins transport zinc out from the cytosol, either to a zinc store or to the extracellular space. To establish which of these zinc transporters are present within oocytes and somatic cells during early folliculogenesis, we first collected pool of primordial, primary, and secondary follicles and performed qPCR to determine the zinc transporters that were expressed in higher amount (Fig. S5*A*). We then performed *in situ* hybridization on the most highly expressed of these target genes including zinc importer genes *Slc39a6*, *Slc39a10* and exporter genes *Slc30a3*, *Slc30a5*, and *Slc30a9* to determine transcript level and subcellular distribution in follicles. Transcript visualization combined with immunostaining of DDX4 (oocyte marker) and Laminin (basement membrane outlining the follicle) allowed definition of mRNA localization relative to oocyte and follicle boundaries (Fig. 5*A*). The puncta number was quantified as total counts in each oocyte and counts per area in the somatic cell region (Fig. 5, *B* and *C*). Among the selected targets, all genes were expressed in the oocyte in the primordial stage at a comparable level. Expression levels of target genes in the oocytes increased significantly at the primary or secondary follicle stages, except for *Slc30a9*, whose expression remained similar during follicle development. At primary and secondary follicle stages, all selected importer genes and the exporter *Slc30a3* were expressed at higher levels than the exporters *Slc30a5* and *Slc30a9* (Fig. S5*B*). On the other hand, the somatic cells exhibited a different profile of zinc transporter expression compared to the oocyte. In pfGCs, transporter targets were expressed at comparable levels between follicle stages (Fig. 5*C*). After somatic differentiation, *Slc39a6* and *Slc30a3* decreased expression significantly in the GCs, while *Slc30a5* and *Slc30a9* increased expression in the primary or secondary follicle stages. In general, the zinc importer and exporter genes increased in expression in the oocytes during folliculogenesis, while in the somatic cells, their expression either decreased or remained at a similar level (Fig. 5, *A–C*). This is consistent with our observation that total zinc content changed to a small degree in somatic cells but increased significantly in the oocyte across these stages of follicle development. Furthermore, we examined the expression of two metallothionein genes, *Mt1* and *Mt2*, which code for the cysteine-rich metal-binding proteins that respond to zinc and act as zinc buffer, by performing *in situ* hybridization on ovarian sections and detected very minimal expression in the oocytes across follicle stages (Fig. S6).

We next examined the protein expression pattern of these selected transporters on ovarian sections using immunohistochemistry. While ZIP6 and ZIP10 were previously reported to be enriched at the plasma membrane of fully grown oocytes (22), we observed minimal plasma membrane staining (magenta arrow) of ZIP6 and ZnT3 in the oocytes of primary to secondary follicle stages (Fig. 5, *D*, vii, *E* and *I*). Most ZIP6 signal was observed in the cytosol (yellow asterisk) and the nucleus (magenta asterisk) of oocytes and somatic cells from primordial to secondary follicles (Fig. 5*D*, i, vii). ZIP10 showed a strong nuclear localization and partial cytosolic pattern in the oocytes and the somatic cells of all follicle stages but was barely detected at the plasma membrane of the oocyte (Fig. 5*D*, ii, viii). Both ZnT3 and ZnT5 showed a prominent nucleolar pattern (yellow arrow), which colocalized with the staining of a nucleolar marker, fibrillarin (Fig. 5*E*). ZnT3 localized to the nucleoli of oocytes, whereas ZnT5 was expressed in the nucleolus of both the oocyte and the somatic cells. Similar to ZIP10, ZnT5 showed clear nuclear staining in the oocyte and both nuclear and cytosolic pattern in the somatic cells. Finally, despite having lower gene expression level in the oocyte and higher in the somatic cells, ZnT9 showed higher cytosolic signal in the oocyte than in the somatic cells. Overall, the dynamic expression of zinc transporters during primordial to secondary follicle development reflects a cell-type–specific regulation of zinc and further suggests subcellular compartmentalization of zinc in the oocyte.

### Perturbation of zinc homeostasis affects somatic survival and proliferation and prevents follicle development

**Figure 6:**
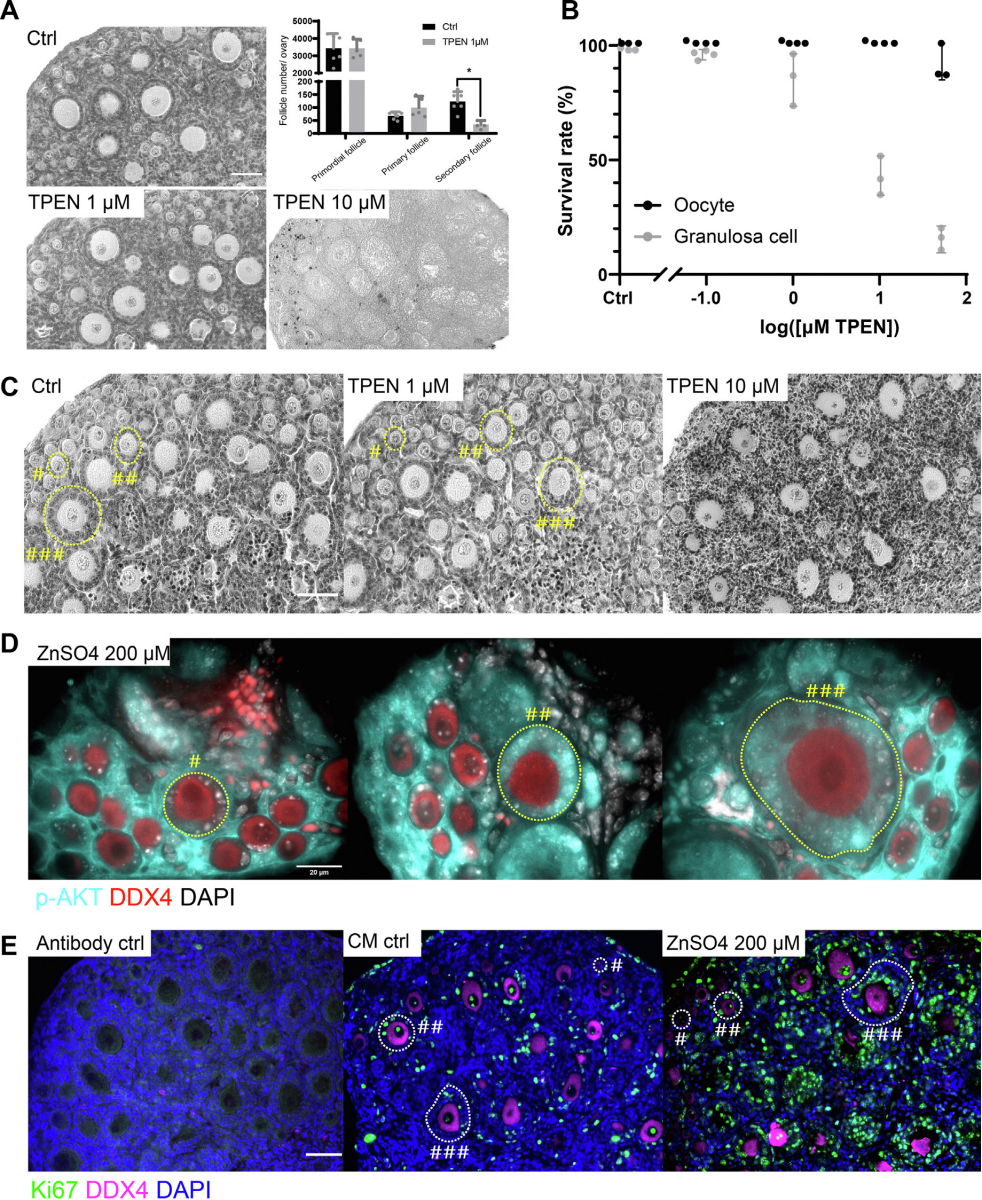
**Zinc manipulation alters somatic survival, proliferation, and secondary follicle development.***A*, H&E staining of P6 ovaries treated with 1 μM or 10 μM TPEN for 24 h followed by 6 days *ex vivo* culture. Ovaries treated with 1 μM TPEN and control ovaries were further counted for follicle number. The scale bar represents 50 μm. *B*, quantification of oocyte and somatic survival rate by live dead staining after 24-h TPEN treatment. *C*, H&E staining of P6 ovaries treated with 1 μM or 10 μM TPEN for 24 h. The scale bar represents 50 μm. *D*, confocal images of phospho-AKT expression analyzed by immunofluorescence staining on the sections of exogenous zinc-treated P6 ovaries. The three panels show different follicle populations under the same treatment condition. p-AKT is shown in *cyan*, DDX4 (oocyte marker) is shown in *red*, with DNA shown in *gray*. See Fig. S5*B* for control staining. The scale bar represents 20 μm. *E*, immunofluorescence images of Ki-67 staining on the sections of exogenous zinc-treated P6 ovaries. Antibody control denotes the technical control group with only secondary antibody, CM control stands for the experimental control with ovaries cultured in culture medium without exogenous zinc. Ki67 is shown in *green*, DDX4 (oocyte marker) is shown in *magenta*, with DNA shown in *blue*. The scale bar represents 50 μm. Data represent mean values (SD). N = 5 to 6 ovaries (*A*) and 3 to 4 repeats of pools of 10 to 20 oocytes and 50 to 100 GCs. Unpaired *t* test was performed for statistically significant differences (*p* = 0.0257) in (*A*). #Primordial follicle, ##Primary follicle, ###Secondary follicle (*C–E*). GC, granulosa cell.

To test whether zinc accrual is necessary for early folliculogenesis, we treated postnatal day (P) 6 ovaries with the zinc chelator N,N,N′,N′-tetrakis-(2-pyridylmethyl)-ethylenediamine (TPEN) (5) for 24 h followed by 6 days of culture without TPEN, to compare how short-term zinc insufficiency affects *in vitro* follicle growth and follicle dynamics. Ovaries treated with 1 μM of TPEN appeared morphologically comparable to controls. Comparison of follicle counts between untreated and treated groups revealed no change in the number of primordial and primary follicles but a significant decrease in secondary follicle number for ovaries in the 1 μM TPEN group (Fig. 6*A*), suggesting a role of TPEN in perturbing follicle progression. Ovaries treated with 10 μM TPEN were significantly smaller than controls and contained no viable follicles. These results demonstrate that zinc homeostasis is necessary for folliculogenesis. To test whether this zinc limitation impacted the oocytes or somatic cells, we employed markers of apoptosis in the ovarian sections after the 24-h TPEN treatments. The primordial, primary, and secondary stage follicles, as well as the interstitial cells, all showed a condensed hematoxylin staining pattern (Fig. 6*C*) with significantly higher TUNEL signal (Fig. S7*A*) in the 10 μM TPEN group; the control and 1 μM TPEN groups were very similar. The oocytes did not seem to be affected morphologically right after 24 h 1 μM TPEN treatment.

To better understand whether oocytes or somatic cells were more sensitive to zinc limitation, we dissociated and isolated these cells from growing follicles and examined cell viability in response to TPEN in doses ranging from 0.1 μM-50 μM. In the 1 μM treatment condition, partial cell death measured by a commercial cell viability assay was observed in the somatic cells (Fig. 6*B*), and when cultured on low-attachment plate, the cells were mostly viable and reaggregated (Fig. S6*C*). In the 10 μM treatment condition, the GCs were not able to reaggregate at the survival rate less than 50% (Fig. S7, *B* and *C*). In contrast to somatic cells, isolated oocytes remained viable when exposed to TPEN at concentrations of 0.1 to 10 μM. While most somatic cells did not survive 50 μM TPEN treatment, more than 90% of oocytes survived this same exposure and remained arrested in prophase of meiosis I with an intact GV (Fig. 6*B*). They did not undergo GV breakdown, the first indicator of meiotic resumption, as fully grown oocytes treated with 10 μM TPEN do (23). These findings demonstrate that oocytes within growing follicles have not yet acquired capacity to resume meiosis.

To determine if specific signaling pathways governing follicle cell survival were responsive to changes in zinc availability, we performed phospho-array analysis in neonatal ovaries after short-term zinc exposure. P6 ovaries were collected and incubated in follicle culture medium with or without 200 μM ZnSO_4_ for 4 h. Phosphorylation ratio (phosphorylated/total protein level) of targets in signaling pathways, as well as a direct comparison of the level of phosphorylated targets, are shown for zinc-treated ovaries and control in Fig. S8*A*. First, we note that this zinc treatment did not activate general stress responses: pHSP90 and p-IKK and p-IKB levels dropped. The short-term zinc exposure led to significant p-AKT activation in the interstitial cells as well as the somatic cells of partial primary follicles and secondary follicles, but not in primordial follicles (Figs. 6*D* and S8*B*). AKT activation following zinc treatment has been reported in human and murine cell lines (24, 25). Our results suggest that p-AKT signal can be detected in primary tissue upon *ex vivo* zinc treatment and that the activation pattern was cell-type (somatic *versus* germ cell) and cell-stage (proliferating *versus* mitotically arrested somatic cell) specific. Given that AKT signaling pathway in the GCs has been reported to regulate somatic survival and proliferation (26, 27), we investigated the expression of Ki-67, a cellular marker for proliferation in zinc-treated ovaries. We observed significant higher Ki-67–positive GCs in primary and secondary follicles of ovaries undergoing short-term zinc exposure (Figs. 6*E* and S8*C*). Together with the zinc limitation results, these experiments establish that proliferation and survival of GCs in growing follicles require accumulation of zinc up to a fixed quota that stays within a narrow range as the follicle develops, whereas the oocyte appears to be more tolerate to a greater degree of zinc modulation.

### Targets in the ubiquitination pathway are enriched in the oocyte as potential zinc-binding partners

**Figure 7:**
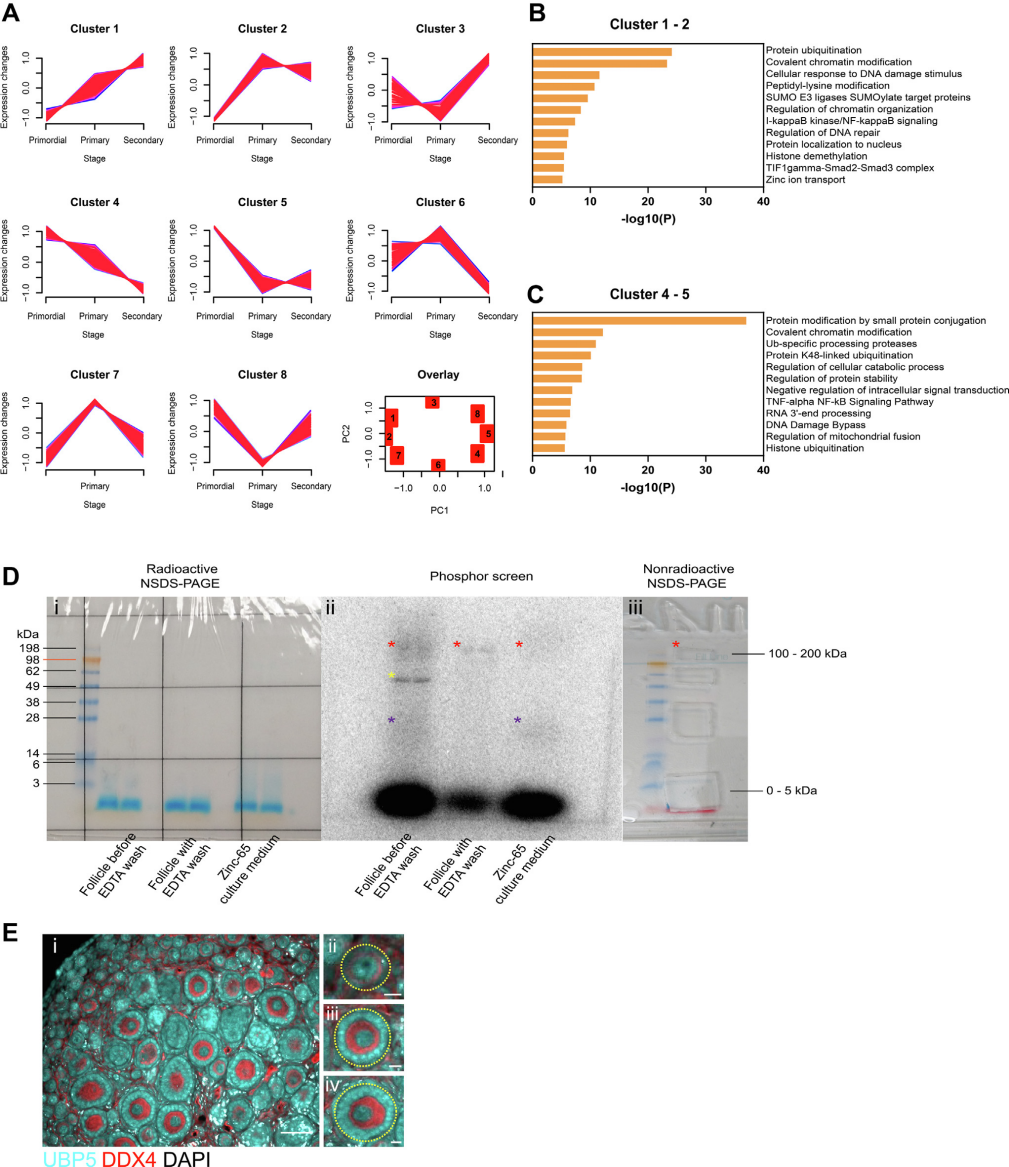
**Targets in the ubiquitination pathway are enriched in the oocyte as potential zinc-binding partners.***A*, differential expression patterns obtained by soft clustering of total 985 unique zinc-related target genes identified in the oocytes across primordial, primary, and secondary follicular stages. Membership values are color-coded with *red* showing high association and *blue* showing low association. Principle component analysis shows relationships between the eight clusters identified. *B*, gene ontology analysis performed on genes increased expression in the oocyte during follicle development (cluster 1, 2). *C*, gene ontology analysis performed on genes decreased expression in the oocyte during follicle development (cluster 4, 5). *D*, native SDS-PAGE image of proteins extracted from follicles cultured in Zn-65 (i). Image of a phosphor screen exposed to the dried PAGE for 5 days before imaging (ii). The two images show Zn-65 signals from follicles with and without EDTA washes, along with the background culture medium. In parallel, protein sample from follicles cultured in nonradioactive zinc was separated, and gel bands with sizes that showed positive Zn-65 signal on the phosphor screen were extracted for protein ID (iii). Asterisks in different colors denote Zn-65–positive protein bands of different sizes. *E*, immunofluorescence images of UBP5 expression on the sections of P6 ovaries. UBP5 is shown in *cyan*, oocyte marker DDX4 is shown in *red*, and DNA in *gray*. Insets from ii to iv show follicles from primordial, primary, and secondary stages, respectively. The scale bar represents 50 μm for i and 10 μm for ii to iv.

The rapid increase of zinc in the oocyte combined with the necessity of zinc homeostasis in follicle development led us to investigate potential zinc-binding targets. We first performed an *in silico* analysis of transcriptomic data on isolated oocytes from primordial to secondary follicles (28) and compared it with all “zinc related genes” obtained from the Uniprot Knowledgebase, which is comprised of 2116 genes (Supplementary File). In total, 985 zinc-related target genes were identified in the oocytes from primordial to secondary follicles. The expression profile of these genes could be clustered into eight different patterns by soft clustering analysis (Fig. 7*A*). Among the clusters that increase (Cluster 1–2) expression throughout oocyte development, many targets were RING motif–containing proteins or E3 ligases in the ubiquitination proteasome pathway that participate in signaling pathways and chromatin modification (Fig. 7*B*). Moreover, zinc transporter genes including *Slc30a5*, *Slc39a6*, and *Slc39a10* were also among the ones with increased expression during oocyte development (Supplementary File). Zinc-related genes that decreased expression (Cluster 4–5) throughout oocyte development contained a different set of E3 SUMO or ubiquitin ligases (Fig. 7*C*).

To verify the transcriptomic results and to identify potential zinc-binding partners in growing follicles, we performed autoradiography and LC-MS/MS on follicles cultured in zinc-containing media. P12 primary and secondary follicles were cultured in Zn-65–containing medium or nonradioactive zinc control medium before rinsing with EDTA to remove excess extrafollicular zinc and extracting for protein separation on a native SDS-PAGE (NSDS-PAGE) (29). The gel containing Zn-65 samples was exposed against a phosphor screen for the identification of Zn-65–positive bands. Meanwhile, the gel bands of samples with potential zinc-binding signal but not exposed to radioisotopes were characterized by LC-MS/MS (Fig. S9). Zn-65 medium after follicle culture, follicles before EDTA wash, and follicles with EDTA wash were lysed and ran on an NSDS-PAGE (Fig. 7, *B* and *I*), followed by exposing and developing on a phosphor screen (Fig. 7*B*, ii). All three sample types showed clear and bright Zn-65 signal at dye front, potentially due to partial denaturation of proteins in the wash buffer which contained 0.02% SDS. In addition, culture medium and follicles before EDTA wash showed two diffused bands, one with molecular weight around 100 to 200 kDa (red asterisk) and the other band around 15 to 30 kDa (purple asterisk). In addition, follicles before EDTA wash showed another clear band at molecular weight around 55 kDa (yellow asterisk). For follicles rinsed with EDTA after Zn-65 culture, only the band at 100 to 200 kDa was spotted. To identify individual zinc-binding targets, we cultured the same stage of follicles in nonradioactive zinc-containing medium, rinsed the follicles with EDTA, ran the NSDS-PAGE with exact experimental settings, and extracted gel band at molecular weight of 100 to 200 kDa for protein ID. In total, 32 unique targets were identified, with 10 targets having the molecular weight ranging in between 100 to 200 kDa (Table S5).

This list of protein targets was then compared with the Uniprot Knowledgebase zinc-related genes in combination with the AlphaFold protein structure prediction database (30) and the web-based zinc-binding site prediction tool, ZincBindPredict (https://zincbind.net/predict/) (31), for zinc-binding site identification. Among the 32 targets, ubiquitin carboxyl-terminal hydrolase 5, also called deubiquitinating enzyme 5 (UBP5), was identified to contain multiple potential zinc-binding motifs including a C3H1 zinc finger domain (Table S6 and Fig. S10). A gel band at dye front with a molecular weight of 0 to 5 kDa was also analyzed for protein identification. Only two unique targets, RS6 and ANXA2 were identified (Table S5). They do not contain a potential zinc-binding motif.

The expression of UBP5 was further confirmed by immunofluorescence staining on P6 ovarian sections. UBP5 showed nuclear expression in the oocytes and the somatic cells of follicles of all stages (Fig. 7*C*). UBP5, encoded by *Usp5* gene, is a well characterized zinc-binding protein: structural studies (32, 33) reveal an N-terminal zinc finger domain which is required for the protein’s catalytic activity. It has been reported to regulate cell proliferation and cell survival in several cancer cell lines and has an increased protein expression during oocyte maturation (34); however, its function in early oocytes is not understood and warrants future investigation. Overall, we identified an enrichment of zinc-binding targets in the ubiquitination pathway that was dynamically regulated in the oocyte during follicle development. We further applied autoradiography and proteomic approaches and identified a deubiquitinating enzyme that potentially binds zinc upon zinc uptake in the growing follicles, providing a mechanistic role for the increase in zinc, namely loading of zinc target proteins that participate in the pathways required for follicle development.

## Discussion

These results establish that zinc is temporally regulated during follicle development. The largest changes in zinc content at the cellular level occur in the oocyte as it transitions from the primordial follicle stage and begins rapid growth. For a dormant oocyte in a murine primordial follicle to develop into a fully-grown oocyte, the largest cell of the organism, there is more than 300-fold increase in oocyte volume and total RNA content, accompanied by a 38-fold increase in the absolute protein synthesis rate (35, 36, 37). During this process, we find that this cell actively accrues more than 30 billion zinc atoms, as demonstrated by both XFM on dried samples and radioactive Zn-65 labeling on live samples. Later in oocyte maturation, the GV oocyte must accrue an additional 20 billion zinc atoms (5). Both major zinc uptake episodes occur over narrow development windows (*i.e.*, 1–2 days).

The increase in zinc in the oocyte is especially profound as it is not just a function of increase in the cell size, but the concentration increases for nearly an order of magnitude when comparing a secondary follicle stage to a primordial follicle stage oocyte. The same level of increase is not observed in other trace metals such as iron and copper, neither in the immediate surrounding somatic cells. The rapid increase in zinc bolus in the oocyte across the timeline of days (primordial follicle is the only class of follicle present in a P0-P1 mouse ovary, by P5-P6, there are primary and secondary follicles present) raises the question of how these cells avoid toxic effects of high zinc concentrations. *In vitro* experiments have demonstrated cell death caused by exogenous zinc treatment through reactive oxygen species and the activation of MAPK pathway (38). This is, however, not observed in the ovary under physiological conditions. Moreover, the expression of metallothioneins, cysteine-rich metal-binding proteins that are typically expressed in response to zinc and act as zinc buffer that controls cytosolic zinc fluctuation, remains at a basal level in the oocytes of primordial to secondary follicular stages, suggesting an efficient transporter-mediated sequestration of labile zinc into intracellular zinc stores. Visualization of labile zinc pools using ZincBY-1 reveals ER and Golgi to be the potential zinc stores of primordial follicle oocyte, and as the follicle develops, this colocalization signal decreases in the oocyte. Studies from our group and others have shown that labile zinc in GV and MII oocytes is stored in cortical granules (39, 40), which are secretory organelles derived from small vesicles of Golgi beginning during primary follicle stage. We therefore propose a model that involves packaging of labile zinc into cortical granules of immature oocytes begins as early as the primary follicle stage. These organelles undergo trafficking to the cortical region as the oocyte matures from GV to MII and eventually are the source of billions of zinc ions released with enzymes that trigger zona reaction upon fertilization where they contribute to blocking polyspermy (15, 39, 40, 41).

Zinc movement into, out of, and within cells is known to be mediated by two large families of homologous solute carrier protein families (10). We find that a subset of these zinc transporters, including ZIP6, ZIP10, ZnT3, ZnT5, and ZnT9, are expressed in a stage-specific manner and are potential mediators of the zinc mobilization and trafficking between compartments that we observe in these developing oocytes (Fig. S11). Staining of ZIP6, ZIP10, and ZnT5 revealed cytosolic localization in the oocyte: since these are integral membrane proteins, we anticipate the staining corresponds to vesicular sites. Additional ZIP6 and ZnT3 staining localized to the plasma membrane; while ZIP10, ZnT3, and ZnT5 staining exhibited clear nuclear/nucleolar expression pattern. ZIP6 and ZIP10 are the two most abundant maternally derived zinc transporters that regulate meiotic maturation in the more mature GV oocyte (22). While the localization of these two transporters shift to cell surface during oocyte maturation (22), a plasma membrane signature was only minimally detected for ZIP6 in primary to secondary follicle oocytes. This suggests a potential shift in zinc uptake mechanism from somatic-dependent (through gap junction) to self-sufficient (through surface zinc transporter) in preparation for ovulation when gap junctions are no longer available. Gap junctions can be found in between the oocyte and pfGCs even before a primordial follicle is formed (42). The fact that zinc increase in the oocytes is follicular stage–dependent, rather than a linear increase which correlates with the size of the oocytes, supports the importance of intercommunication between the two compartments through gap junctions, particularly in zinc regulation.

It has been estimated that 30 to 40% of total cellular zinc is present in the nucleus of typical mammalian cells, including the nucleolus (43). So far, only one study has reported nucleolar localization of zinc transporter-ZIP2 in human macrophages (44). Our current work indicates that ZIP10, ZnT3, and ZnT5 could be novel nucleolar zinc transporters in murine oocytes. As nucleolus is a nonmembrane–bound structure, it is more likely that these transporters are located on the vesicles associated with the nucleolus, regulating subregional zinc availability. Our *in silico* transcriptomic analysis revealed dynamic regulation of zinc target genes in the oocyte during follicle development, with many being enriched in the ubiquitination pathway. Interestingly, as ubiquitin and ubiquitin-like proteins actively participate in ribosome biogenesis, several enzymes in the ubiquitination pathway have been reported to regulate nucleolar function (45, 46). Furthermore, autoradiography and protein ID method indirectly identified nuclear UBP5 to be potential zinc-binding targets during the 2-day incubation period. This method was unable to reveal all zinc-binding targets unless performed *in vivo*. Further analysis on the properties of each specific zinc-binding sites is required to support the hypothesis that the (un)loading of zinc from a weak (nonstructural) binding site acts as molecular switches to regulate protein activity directly.

Besides cell cycle stages, specific zinc fluctuations and associated transporter localization have been reported in other developmental transitions. For example, B cells show higher labile zinc level and ZIP7 expression after cell activation, in which state they start proliferating and differentiating (47). This is not observed during the activation of pfGCs. In a primordial follicle, the somatic cells are mitotically inactive until follicle activation, when the cells become cuboidal and mitotically active. Nevertheless, elemental contents in single somatic cells showed high degree of variability indicated by the magnitude of the standard deviation for iron and zinc (Fig. 3*B*). Since GCs undergo cell division asynchronously (48), it is likely that this variation is associated with the mitotic status of the cell. Ki-67 labeling of control ovaries showed positive signal on selective GCs within a follicle, indicating while some somatic cells in a follicle are in active phases of the cell cycle, others remain in the resting G0 phase. Exogenous zinc treatment greatly increased Ki-67–positive GCs within a follicle, recapitulating studies showing cell cycle re-entry after zinc supplementation in Zn^2+^ deficiency–induced quiescent cells (49). The treatment, however, did not increase pfGCs proliferation and differentiation in primordial follicles, suggesting pfGCs proliferation regulated by mTOR (50) and TGFβ- SMAD3 (51) signaling is inherently distinct from GCs proliferation in growing follicles. Several studies have reported the importance of the PI3K-AKT pathway in GCs proliferation and survival (26, 27). As a PI3K/AKT antagonist, PTEN (phosphatase and tensin homolog) activity can be regulated by zinc through indirect proteasome pathway (24) or direct interaction with Cys71 and Cys124 of PTEN (25). Our study suggests a potential zinc requirement in GC proliferation and survival through AKT activation. Intriguingly, the oocyte is significantly more resistant to acute zinc limitation *via* TPEN treatment and zinc excess than somatic cells. One possible explanation is that the burst of zinc uptake in the developing oocyte leads to saturation of many of the essential sites of zinc action including zinc-dependent hydrolytic enzymes, zinc finger transcription factors, ribosomal subunits, and compartments like the secretory pathway vesicles.

Finally, this study sheds light on fluctuations in other metals that serve as essential cofactors. During mammalian folliculogenesis, the number of mitochondria in a primordial follicle oocyte increases from few thousands to more than a hundred thousand in a mature oocyte (52, 53). This potentially explains the increasing requirement of iron and copper particularly in oxygen metabolism and electron transfer. We also find an increase in metal concentration in murine oocytes for manganese which is essential for mitochondrial superoxide dismutase as well as a number of glycan-conjugating enzymes, cobalt, an essential cofactor in vitamin B12, and nickel which has no known role in mammalian biology (Table S2). Our group has recently described a novel manganese exocytosis that occurs alongside zinc efflux that potentially contribute to block to polyspermy during fertilization in *Xenopus* eggs (54). As TPEN also has affinity for other heavy metals including iron and copper (5, 7, 23), further studies are required to investigate the biological roles of these trace elements during oocyte development.

Overall, we describe active zinc accrual and dynamic regulation of zinc during ovarian follicle development. While total zinc content in individual somatic cells remains at a constant level, significant stage-specific increase and compartmental redistribution in oocyte zinc content is observed upon the initiation of early follicle growth, which coincide with increased zinc transporter expression. Upon zinc manipulation, oocytes demonstrate high tolerance to zinc deficiency, while somatic survival and proliferation are sensitive to zinc chelation or supplementation, resulting in decreased secondary follicle number in the ovary. Finally, we performed proteomic and *in silico* transcriptomic analysis to investigate potential zinc targets in the oocyte and identified an enrichment of targets in the ubiquitination pathway. While mechanisms regulating the dynamics of zinc availability in early-staged follicles remain to be discovered, this quantitative physiological analysis establishes zinc as specific, regulated events that instruct early folliculogenesis.

## Experimental procedures

Chemicals, reagents, and buffer were purchased from MilliporeSigma, and all procedures were carried out at room temperature unless otherwise specified. All solutions were prepared using volume concentration (% v/v).

### Animals

Female CD-1 mice were purchased from Envigo and bred in-house at the Northwestern University Center for Comparative Medicine animal facility. Animals were treated in accordance with the National Institutes of Health Guide for the Care and Use of Laboratory Animals. Food (Teklad 2020X, Envigo) and water were given *ad libitum* and the mice were kept in a 14-h light, 10-h dark cycle with constant temperature and humidity. All protocols were approved by the Northwestern University Institutional Animal Care and Use Committee.

### Follicle and single cell isolation

Primordial and primary follicles were isolated from the ovaries of CD-1 postnatal day 6 (P6) mice by three rounds of enzymatic and mechanical digestion (19, 55). Briefly, the ovaries were dissected out of the bursa, then incubated on a 37 °C heated stage for 13 min in Leibovitz's L-15 medium (Thermo Fisher Scientific) supplemented with 1 mg/ml poly(vinyl alcohol) (PVA, hereafter referred to as L-15/PVA), 30.8 μg/ml Liberase TM (Roche), and 456 U/ml DNaseI (Worthington). Following incubation, the ovaries were rinsed in L-15/PVA and then transferred to L-15/PVA supplemented with 50 μl/ml fetal bovine serum (Thermo Fisher Scientific) for mechanical digestion by repeated pipetting for 4 min. The process was then repeated twice more in L-15/Liberase for 7 and 4 min, followed by rinsing and mechanical digestion after each digestion. After the third round, follicles were selected manually using a mouth pipet under a dissection microscope and washed in three drops of L-15/PVA with 100 μl/ml FBS to quench enzymatic activity. Secondary and/or primary follicles were isolated from P12 mice with a mechanical-only digestion method. After being dissected out of the bursa, the ovaries were mechanically disrupted *via* ‘flicking’ with insulin syringe needles (Thermo Fisher Scientific) until their follicles were released. Individual follicles were collected and staged under 40× brightfield objective. Follicles with only one layer of squamous somatic cells were classified as primordial follicles; primary follicles were classified as containing only one layer of cuboidal somatic cells, and secondary follicles were classified as containing exactly two layers of somatic cells.

For single oocyte and somatic cell collection, follicles were staged and then incubated in Accutase solution (Stemcell Technologies) for 5 to 10 min at 37 °C before dissociating somatic cells from the oocytes by mechanical agitation. For GV-stage oocytes collection, 6- to 8-weeks-old CD-1 females were injected intraperitoneally with 5 IU pregnant mare’s serum gonadotropin 48 h prior to the collection. Ovaries were dissected and the oocytes were collected by mechanical agitation of the ovaries in L-15/PVA supplemented with 10 μM milrinone. Collected GV oocytes were then washed briefly in acidic Tyrodes solution to remove the ZP for the subsequent staining.

### Synchrotron-based XFM

Isolated follicles or single cells were washed in 100 mM, pH 7 ammonium acetate solution supplemented with 1 mg/ml PVA to remove metal salts in the media before being placed on an intact 1.5 mm × 1.5 mm silicon nitride window (Norcada Inc). Excess liquid was removed by mouth pipetting. The windows were placed on a 37 °C heated stage for less than 1 minute until dry and stored at room temperature until imaging.

Synchrotron-based XFM was performed at the BNP (18) located at sector 9-ID-B at the Advanced Photon Source of Argonne National Laboratory. 10 keV x-rays were monochromatized double crystal Si(111) monochromator and focused to a spot size of 80 to 100 nm using Fresnel zone plate optics. Raster scans were done in fly-scan mode with continuous motion in the horizontal direction and stepping motion in the vertical direction in steps of 100 to 150 nm for both whole follicles and single cells. Fluorescence spectra were collected using a silicon drift detector (Vortex-ME4) with 30 to 100 msec dwell time per pixel for whole follicles, 30 to 80 msec for single oocytes, and 100 msec dwell time per pixel for single somatic cell scanning.

The fluorescence signals in counts/s were converted to a 2D concentration in μg/cm^2^ by fitting the per-pixel spectra with a modified Gaussian distribution and comparing against an AXO thin-film standard (AXO Dresden GmbH). It was assumed that no elemental content was lost during sample preparation. Elemental quantification and image processing were performed using the MAPS 1.8.0.00 software (56, 57) by drawing regions of interest covering whole follicles or individual cells on the phosphorous channel.

### Zn-65 uptake

Radioactive zinc media was prepared by adding 4 μCi of Zn-65 (National Isotope Development Center) diluted in nonradioactive zinc sulfate by 1:50 total zinc atom number in 0.1 M hydrochloric acid (Thermo Fisher Scientific), into final 1 ml follicle culture medium (1:1 MEMα, GlutaMAX and Ham’s F-12 medium, GlutaMAX with 3 mg/ml bovine serum albumin, BSA. Both media were purchased from Thermo Fisher Scientific). Primordial follicles and growing follicles comprised of primary to early secondary follicles sizing 80 to 100 μm were isolated from P6 and P12 CD-1 mice ovaries, respectively. Around 100 to 500 primordial follicles were first cross-linked by treating with 35 μg/ml phytohemagglutinin (PHA-P) (58, 59) for 30 min at 37 °C; the follicles were pelleted by spinning at 10,000 × g for 1 min and encapsulated in 5 mg/ml alginate (60, 61) for the following treatment. Encapsulated primordial follicles and about 200 growing follicles were incubated in radioactive zinc media for 24 h and 2.5 h, respectively in 37 °C incubator with 5% CO_2_. After incubation, primordial follicles were then collected by incubating the alginate in 10 IU/ml alginate lyase for 15 to 20 min on a 37 °C heat plate. Follicles were than washed in 1 ml of Dulbecco’s phosphate buffered saline (DPBS) with 1 mg/ml PVA and 110 mM EDTA for 4 times before being lysed in 1 ml of 1 % SDS solution. Radioactivity was subsequently measured by the Wizard^2^ gamma counter (PerkinElmer) with 900 s count time, before being converted to atom number using the standard solutions measured on the same day. Background zinc level in the culture medium before adding in Zn-65 was measured using a Thermo Scientific iCAP Q ICP-MS (Quantitative Bio-element Imaging Center, Northwestern University). Briefly, 150 μl of the medium was added in a 15 ml metal-free conical tube with 150 μl 70% nitric acid (Thermo Fisher Scientific) and incubated on a 70 °C heat block for 4 h. H_2_O provided by a Milli-Q Ultrapure Water System was then added to dilute the acid to a final volume of 5 ml for the analysis. All protocols concerning radioactive material storage and handling were approved by Northwestern University Office for Research Safety.

### TPEN treatment and *ex vivo* ovarian tissue culture

Freshly dissected P6 ovaries were placed on a 0.4 μm Millicell cell culture insert and incubated in follicle culture medium supplemented with 0, 1, or 10 μM N,N,N′,N′-tetrakis-(2-pyridylmethyl)-ethylenediamine (TPEN) in 37 °C incubator with 5% CO_2_ for 24 h. While some ovaries were collected and fixed in Modified Davidson’s fixative (Electron Microscopy Sciences) for 16 h at 4 °C for histological analysis, others were transferred to follicle growth medium to culture for another 6 days under the same incubation condition. Follicle growth medium was prepared using 1:1 MEMα, GlutaMAX and Ham’s F-12 medium, GlutaMAX with 3 mg/ml BSA, 1 mg/ml bovine fetuin, 10 mIU/ml recombinant follicle-stimulating hormone, 5 μg/ml insulin, 5 μg/ml transferrin, and 5 ng/ml selenite. recombinant follicle-stimulating hormone was received from A.F. Parlow, National Hormone and Peptide Program, National Institute of Diabetes and Digestive and Kidney Diseases. During the 6-day culture period, 50% of the old medium was replaced with fresh follicle growth medium every other day. At the end of the culture, ovaries were collected for fixation as described above and processed with the following histological analysis.

### Histology and follicle counting

After fixation, ovaries were washed in 50%, 60%, and 70% ethanol (Decon Labs Inc) for 5 min each. Ovaries were then dehydrated using a tissue processor (Leica) before embedding in paraffin blocks (Mercedes Scientific). Serial sections of the whole ovaries were cut at 5 μm (Leica) and let dry at 25 °C. H&E staining was performed with an autostainer (Leica) using the standard protocol. Follicles showing clear nuclear (primordial follicle) or nucleoli (growing follicle) staining were counted on every fifth section with classification criteria as shown in Figure 1*A*. The sum of each follicle class was then averaged for the counted sections, then multiplied by total section number to obtain total follicle number per ovary. To account for duplicating the count of small follicles, total follicle number of primordial and primary follicles was further divided by 2.

### Live dead assay and GC reaggregation

Oocytes and somatic cells of secondary follicles were isolated from P12 mouse ovaries as described above. Isolated oocytes and somatic cells were placed on a flat bottom 96-well cell culture plate (Corning Inc) with follicle culture medium supplemented with different concentrations of TPEN and cultured in a 37 °C incubator with 5% CO_2_ for 24 h. Cell survival was then quantified by adding the Live and Dead Dye (Abcam) at a final concentration of 5X in DPBS/1 mg/ml PVA at 25 °C for 10 min in the dark. Cells were then imaged by the EVOS cell imaging system, with live cells detected using the GFP channel and dead cells detected by the RFP channel. To measure somatic cell reaggregation, instead of using a regular flat bottom plate, somatic cells were placed on a round bottom, ultra-low attachment 96-well plate (Corning Inc) and incubated under the same conditions for 24 h before imaging.

### Phospho-array experiment

Eight P6 single mouse ovaries were collected and incubated in follicle culture medium with or without 200 μM ZnSO_4_ for 4 h in a 37 °C incubator for each group. The ovaries were then snap frozen in liquid nitrogen and stored at −80 °C until use. Cell signaling phospho antibody array (Full Moon BioSystems) was used for the experiment. The tissue was digested according to the manufacturer’s recommendations. Protein quality was measured using the NanoDrop 1000 (Thermo Fisher Scientific) and 150 OD of protein was used for biotinylation for each condition. The arrays were imaged and analyzed by the manufacturer.

### Immunofluorescence staining and TUNEL assay

Ovarian paraffin sections prepared as described above were deparaffinized using the standard protocol. Antigen retrieval was then performed by boiling the slides in 10 mM sodium citrate, pH 6.0, with 0.05 % Tween-20 using a pressure cooker (Maxi-Matic Elite Platinum) for 30 min. After cooling off, the slides were then blocked in DPBS with 2 % donkey serum (Jackson ImmunoResearch), 0.1 % fish skin gelatin, 10 mg/ml BSA, 0.1 % Triton X-100, 0.05% Tween-20, and 0.05 % sodium azide (w/v) for 1 h, followed by primary antibody incubation at 4 °C for 16 h. Primary antibodies (Table S7) were prepared in the above blocking buffer with dilutions described in the table. We generated the ZIP6, ZIP10, and ZnT5 homemade antibodies (22), while others were commercially available antibodies. The specificity for the commercially available antibodies was not further confirmed in loss of function models. Slides were washed three times in 1× PBST for 5 min each time, before being incubated in secondary antibodies for 1 h. Secondary antibodies were prepared at the dilution of 1:200 in blocking buffer. Slides were washed 3 times in 1X PBST for 5 min each time, followed by 1× PBS wash for 5 min, before mounted in DAPI-containing antifade mounting medium (Vector Laboratories) for imaging. TUNEL assay was performed using the DeadEnd Fluorometric TUNEL System (Promega) with the provided manufacturer’s protocol.

### Labile zinc and compartment costaining

Isolated follicles or oocytes were stained with 100 nM ZincBY-1 in L-15/PVA medium on 37 °C heated stage for 30 min. Hoechst 33342 (Thermo Fisher Scientific) was added at 1 μg/ml 10 min before the ZincBY-1 staining was completed. Cells were then washed in three drops of 50 μl L-15/PVA and transferred to a 20 μl droplet of fresh L-15/PVA medium on a 35 mm glass bottom dish (MatTek Corporation) covered with mineral oil and imaged with a confocal microscope. For ER staining, the follicles were incubated with 1 μM ER tracker (Invitrogen) and 100 nM ZincBY-1 in L-15/PVA medium on 37 °C heated stage for 30 min. For Golgi labeling, follicles or oocytes were incubated in 5 μM BODIPY-TR–labeled sphingolipids (Invitrogen) at 4 °C for 30 min following ZincBY-1 staining. For mitochondria staining, follicles were incubated in 200 nM MitoTracker Red CMXRos (Thermo Fisher Scientific) and 100 nM ZincBY-1 in L-15/PVA medium on 37 °C heated stage for 30 min. Hoechst 33342 was added 10 min before the last step of the staining and prepared for confocal imaging as described.

### Real time - PCR

Around 100 fresh primordial follicles, 20 primary, and 20 secondary follicles were isolated and lysed directly in 50 μl extraction buffer provided in the Arcturus PicoPure RNA isolation kit (Thermo Fisher Scientific), snap frozen in liquid nitrogen, and stored at −80 °C until use. RNA isolation was performed according to the manufacturer’s recommendations with on-column DNaseI treatment. Eluted RNA was then reverse-transcribed using the Superscript III first-strand synthesis super mix (Thermo Fisher Scientific). Real time-PCR was performed using the SYBR Green master mix (Thermo Fisher Scientific) and primers (Integrated DNA Technologies) (Table S8) on the StepOnePlus real-time PCR system (Applied Biosystems) with 56 °C annealing temperature and 40 run cycles. Relative expression level of each target genes was calculated using the 2^−ΔΔCt^ method normalized to the expression level of GAPDH. Data was pooled from experiments performed on at least three independently collected biological samples.

### RNA *in situ* hybridization and quantification

CD-1 P6 ovaries were dissected and fixed in 10% neutral buffered formalin at room temperature for 24 h before being processed for paraffin embedding and sectioning into 5 μm slices. We used the Multiplex fluorescent reagent kit v2 (Advanced Cell Diagnostics) for RNA *in situ* hybridization experiment. All procedures were carried out according to manufacturer’s recommendations (Table S9). The specificity of the commercial probes was based on the sequence of the probe. We did not further verify probe specificity. Before DAPI staining, the slides were counterstained with anti-DDX4 (1:100) and anti-Laminin (1:100) antibodies for 1 h at room temperature, followed by anti-HRP antibody (1:200) and Opal-690 (1:750, PerkinElmer) incubation for 30 min and 10 min, respectively. The slides were imaged with a Leica SP5 confocal microscope (Leica Microsystems). Transcript quantification was performed using particle counting on ImageJ. Follicles were quantified from 6 to 8 different ovaries.

### In silico transcriptomic analysis

Zinc-related genes were downloaded from UniProt Knowledgebase, Swiss-Prot (reviewed and manually annotated), with organism restricted to *Mus musculus* and a search keyword “zinc” was applied. In total, 2116 genes were identified (Supplementary File). The genes were compared with RNA-seq dataset published by Veselovska *et al*.(28) (GSE 70116). Among the dataset, “Non-growing oocytes (NGO)”, “Growing oocyte 1 (GO1)”, and “Growing oocyte 2 (GO2)” were used for the analysis of this study. In terms of clarity, “NGO” group was labeled as “Primordial stage”, “GO1” labeled as “Primary stage”, and “GO2” as “Secondary stage” in the current work. In total, 985 unique target genes were identified in the oocytes across primordial, primary, and secondary follicular stages after filtering out for low expression genes (Supplementary File). Target genes were then subjected to soft clustering using the Mfuzz software package (62, 63), with further gene annotation analysis performed on Metascape (64) (Supplementary File).

### Zn-65 autoradiography

Around 100 to 120 growing follicles comprised of primary to early secondary follicles sizing 80 to 100 μm were isolated from P12 CD-1 mice ovaries and incubated in follicle culture medium (as described above) containing 4 μCi of Zn-65 or equal amount of nonradioactive zinc sulfate for 2 days in 37 °C incubator with 5% CO_2_. Follicles were than washed in 1 ml DPBS with 1 mg/ml PVA and 110 mM EDTA for 4 times before transferring to a 1.5 ml Eppendorf tube with 300 μl Milli-Q water. The tube was repeatedly freeze-thawed in liquid nitrogen and room temperature Milli-Q water for a total of three cycles and then sonicated in a water bath (SonicsOnline) for 2 min. The sample solutions were then concentrated using 0.5 ml 3K Amicon ultra centrifugal filters and spun at 14,000 × rpm for 15 min at room temperature. The volume of the final concentrated solutions was about 40 μl. Radioactive and nonradioactive samples were run on separate gels for autoradiography film exposure and protein ID, respectively. Briefly, SeeBlue Plus2 prestained protein standard (Thermo Fisher Scientific) and follicle sample with nondenaturing sample buffer (final concentration 20 mM Tris–HCl, 37.5 mM Tris-Base, 2.5% glycerol, 0.01875% Coomassie blue, 0.000625% phenol red in Milli-Q water, pH= 8.0) were ran on NSDS gels (29) in running buffer 50 mM MOPS, 50 mM Tris–Cl, and 0.02% SDS, pH= 7.3 for 150v 60 min. NSDS gels were prepared with 12% resolving bottom gel (4× Bis–Tris (1.5 M, pH= 6.7) 2 ml, SureCast 40% w/v Acrylamide (Thermo Fisher Scientific) 2.4 ml, 10% APS 80 μl, TEMED 8 μl, H_2_O 3.5 ml) and 12% stacking upper gel (4× Bis-lTris 250 μl, SureCast 40% w/v Acrylamide 100 μl, 10% APS 10 , TEMED 1 μl, H_2_O 639 μl).

Gels containing radioactive sample were then sandwiched in between two cellophane sheets (Thermo Fisher Scientific) presoaked with gel drying solution (Thermo Fisher Scientific) and dried for 16 h. The dried gels were then exposed against a BAS-IP MS Phosphor Screen (GE Healthcare) at room temperature for 5 days before being developed using a Typhoon phosphorimager (GE Healthcare). Alternatively, gels containing nonradioactive sample were cut at their corresponding sample sites that showed positive signal on the radioactive gel. Gel bands were washed in 100 mM Ammonium Bicarbonate (AmBic)/Acetonitrile (ACN) and reduced with 10 mM DTT at 50 °C for 30 min. Cysteines were alkylated with 100 mM iodoacetamide in the dark for 30 min in room temperature. Gel bands were washed in 100 mM AmBic/ACN prior to adding 600 ng trypsin for overnight incubation at 37 °C. Supernatant-containing peptides were saved into new tubes. Gels were washed at room temperature for 10 min with gentle shaking in 50% ACN/5% formic acid (FA), and supernatant was saved to peptide solution. Wash steps were repeated each by 80% ACN/5% FA and 100% ACN, and all supernatant was saved then subject to the speedvac dry. After lyophilization, peptides were reconstituted with 5% ACN/0.1% FA in water and injected onto a trap column (150 μm ID × 3 cm in-house packed with ReproSil C18, 3 μm) coupled with an analytical column (75 μm ID × 10.5 cm, PicoChip column packed with ReproSil C18, 3 μm) (New Objectives, Inc). Samples were separated using a linear gradient of solvent A (0.1% FA in water) and solvent B (0.1% FA in ACN) over 60 min using a Dionex UltiMate 3000 Rapid Separation nanoLC (Thermo Fisher Scientific). MS data were obtained on an Orbitrap Elite Mass Spectrometer (Thermo Fisher Scientific). Data were searched using Mascot (Matrix Science) v.2.5.1 against the Swiss-Prot Mouse database, and results were reported at 1% FDR in Scaffold v.4.8.4 (Proteome Software). Targets identified were compared to a list of zinc-related genes downloaded from UniProt Knowledgebase (Supplementary File).

### Statistical analysis

Elemental contents of follicles and single cells, as well as zinc transporter expression, were analyzed for statistical significance using two-way ANOVA with Tukey’s or Śídák’s multiple comparison tests. *p* < 0.05 was considered statistically significant. Zn-65 experiments and follicle quantification were analyzed by unpaired *t* test. All statistical tests were performed using GraphPad Prism 9 (GraphPad Software).

## Data availability

All data are contained within the article.

## Supporting information

This article contains supporting information.

## Conflict of interests

The authors declare that they have no conflicts of interest with the contents of this article.
