# Supplementary material for: Zinc dynamics regulate early ovarian follicle development
Source: J Biol Chem. 2022 Nov 22;299(1):102731. doi: 10.1016/j.jbc.2022.102731 (PMC9800340; doi:10.1016/j.jbc.2022.102731)
Supplement: Supplemental Figures S1–S11 and Tables S1–S9 [file mmc2.docx]

**Supplementary Legends**

**Figures**

**Supplementary Figure 1**. Total sample area of whole follicles (A), oocytes (B) and single somatic cells (C) quantified from the 2D scan images of the phosphorous channel on the MAPS software.

**Supplementary Figure 2**. Primordial and growing follicles were incubated in Zn-65 for 24 and 2.5 hours respectively, followed by washes in PBS/ EDTA for 4 times (wash 1- 4) before being lysed in SDS (Follicles). The radioactivity in the media, buffer, and follicle samples for primordial (A) and growing follicles (B) were measured by a gamma counter as counts per minutes (CPM). The counts were then converted into atom number per follicle in the two groups (C).

**Supplementary Figure 3**. Distribution of zinc in the oocytes and the somatic cells in whole follicles in percentage (A) and total atom number (B) were calculated by subtracting the averaged zinc content in the oocytes from the averaged zinc content in whole follicles of respective stages (The left panel in B shows the unscaled versus the scaled right panel for visualization).

**Supplementary Figure 4**. Labile zinc co-staining with mitochondria in the follicles. Primordial and primary follicles collected from P6 ovaries were stained with ZincBY-1 (green) and mito-tracker (magenta) to determine the colocalization of labile zinc in the mitochondria. Hoechst indicates DNA staining. Scale bar= 10 μm.

**Supplementary Figure 5**. Zinc transporter gene expression in the follicles and individual cells. Primordial and primary follicles isolated from P6 ovaries, and secondary follicles isolated from P12 ovaries were measured for normalized zinc transporter gene expression by real time- PCR (A). Letters denote statistically significant differences between different transporter genes within each follicle stages by one-way ANOVA with Tukey’s multiple comparison test (p<0.05). Expression level of selected targets including *Slc39a6*, *Slc39a10*, *Slc30a3*, *Slc30a5*, *Slc30a9* were further quantified on ovarian section by in situ RNA hybridization. B and C show the remakes of Figure 5B, C by grouping the follicle stages together. Data represent mean values (SD).

**Supplementary Figure 6**. The expression of metallothionein genes in the follicles. *Mt1* and *Mt2* expression levels were measured by *in situ* RNA hybridization on non-treated (control) versus 4-hr 200 μM ZnSO_4_ treated P6 ovarian section, which served as a technical positive control as metallothionein genes increase expression upon zinc treatment. The red puncta represent the amplification of each *Mt* transcripts, while the gray marks the boundary of the oocyte (DDX4) versus the somatic cells (basement membrane of the follicle). Scale bar= 20 μm.

**Supplementary Figure 7**. The effect of 24-hour TPEN treatment was manifested on somatic apoptosis as shown by TUNEL staining on a P6 ovary (Red color indicates TUNEL positive signals, blue indicates DNA. Scale bar= 50 μm) (A). Live dead staining on individual somatic cells and oocytes confirmed higher sensitivity to zinc deprivation in the somatic cells than in the oocytes (Live cells are shown in cyan, dead cells are shown in red. Scale bar= 100 μm) (B). The somatic granulosa cells lost the ability to reaggregate after 24-hour 10 μM TPEN treatment (Scale bar= 200 μm) (C).

**Supplementary** **Figure 8**. Short-term zinc exposure promotes AKT activation in the somatic cells. P6 ovaries were collected and incubated in follicle culture medium with or without 200 μM ZnSO_4_ for 4 hours for phospho- array analysis. Red dots indicate phosphorylation signal that increased after zinc exposure, while blue dots indicate decreased phosphorylation signal (A). P-AKT expression was confirmed with immune staining on P6 ovarian section with technical control (staining without primary antibody), experimental control (culture medium without zinc treatment), and 200 μM ZnSO_4_ treated groups (p-AKT is shown in cyan, DDX4, the co-stained oocyte marker is shown in red, with DNA shown in gray. Scale bar= 100 μm) (B). Significant higher Ki-67 signal was observed in GCs of primary and secondary follicles of ZnSO_4_ treated ovaries. The signal was quantified by the percentage of Ki-67-positive GCs within each growing follicle (C) (N= 15-20 follicles). Data represent mean values (SD). Unpaired t test was applied for computing statistically significant differences between control and treatment groups (***p<0.001).

**Supplementary Figure 9.** Illustration of autoradiography and LC-MS/MS on growing follicles isolated from P12 ovaries. Follicles were incubated with or without Zn-65 containing culture medium for 48 hours, washed with EDTA before lysed for protein extraction, gel separation and protein ID.

**Supplementary Figure 10.** Potential zinc binding sites of mouse UBP5 (UniProtKB accession code: P56399) performed by in combination of a three-dimensional protein structure obtained from the AlphaFold protein structure prediction database and a web-based zinc binding site prediction tool, ZincBindPredict (<https://zincbind.net/predict/>). The spheres imbedded in the protein ribbon diagram present residues of predicted zinc binding sites (white ribbon: predicted mouse UBP5 structure [amino acid sequence: 1-858], magenta spheres: predicted C3H1 zinc binding site [UBP-type zinc finger domain]). The red-dotted boxes display enlarged predicted zinc site with rod-shaped residues (yellow parts: sulfur, blue parts: nitrogen, magenta: carbon atoms of C3H1 ZF site). A black dotted gray sphere in the red-dotted box is a presentation of a possible zinc ion bound to the predicted sites.

**Supplementary Figure 11.** Summary diagram of zinc regulation in early follicles. Colors from beige to brown indicate lower to higher total zinc concentration, with labile zinc ions shown in red circles.

**Tables**

**Supplementary Table 1**. Summary of elemental concentration and total atom number of all measured elements in primordial, primary, and secondary follicles. Elemental concentration is the mean value in the unit of μg/cm^2^ after subtracting the background, while total atom number is the multiplication of concentration and the ROI which represents total sample area. All values are shown in mean (SD). (Primordial follicle N=10, primary follicle N=8, secondary follicle N=6)

**Supplementary Table 2**. Summary of elemental concentration and total atom number of all measured elements in oocytes of primordial, primary, and secondary stage follicles. Elemental concentration is the mean value in the unit of μg/cm^2^ after subtracting the background, while total atom number is the multiplication of concentration and the ROI which represents total sample area. All values are shown in mean (SD). (Primordial follicle oocyte N=5, primary follicle oocyte N=12, secondary follicle oocyte N=11)

**Supplementary Table 3**. Summary of elemental concentration and total atom number of all measured elements in somatic cells of primordial, primary, and secondary stage follicles. Elemental concentration is the mean value in the unit of μg/cm^2^ after subtracting the background, while total atom number is the multiplication of concentration and the ROI which represents total sample area. All values are shown in mean (SD). (Primordial follicle somatic cell N=17, primary follicle somatic cell N=14, secondary follicle somatic cell N=10)

**Supplementary Table 4**. Summary of correlation coefficient (Pearson’s R value computed with coloc 2) of labile zinc staining with ER and Golgi-tracker. All values are shown in mean (SD). Statistically significant difference between GV and primordial stages was computed by one-way ANOVA with Tukey’s multiple comparison test (**p < 0.01, ****p < 0.0001).

**Supplementary Table 5.** List of proteins identified from autoradiography and protein ID.

**Supplementary Table 6.** Mouse UBP5 (UniProtKB accession code: P56399) zinc binding site prediction results using ZincBindPredict, a web-based zinc binding site prediction tool (<https://zincbind.net/predict/>). The predicted 3D structure of mouse UBP5 was obtained from the AlphaFold protein structure database and utilized to predict potential zinc binding sites. The Precision Score presents how effective at ignoring non-binding sites (max. 1.00). Total 51595 sites were considered as a zinc binding site by ZincBindPredict, and 51581 sites were rejected. Finally, 9 sites were selected as potential zinc binding sites (duplicated sites were excluded).

**Supplementary Table 7**. List of antibodies used for immunofluorescence staining.

**Supplementary Table 8**. List of primers used for RT-PCR experiment. F denotes forward primers while R denotes reverse primers.

**Supplementary Table 9**. List of probes used for the RNAscope *in situ* hybridization experiment.

**Supplementary Figures**

Supplementary Figure 1


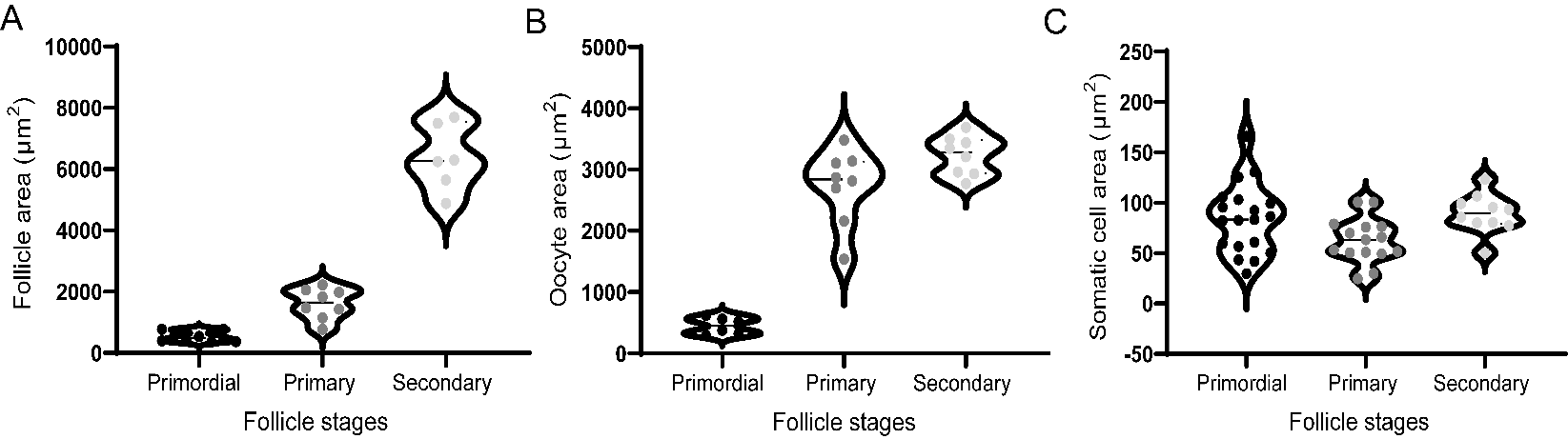


Supplementary Figure 2


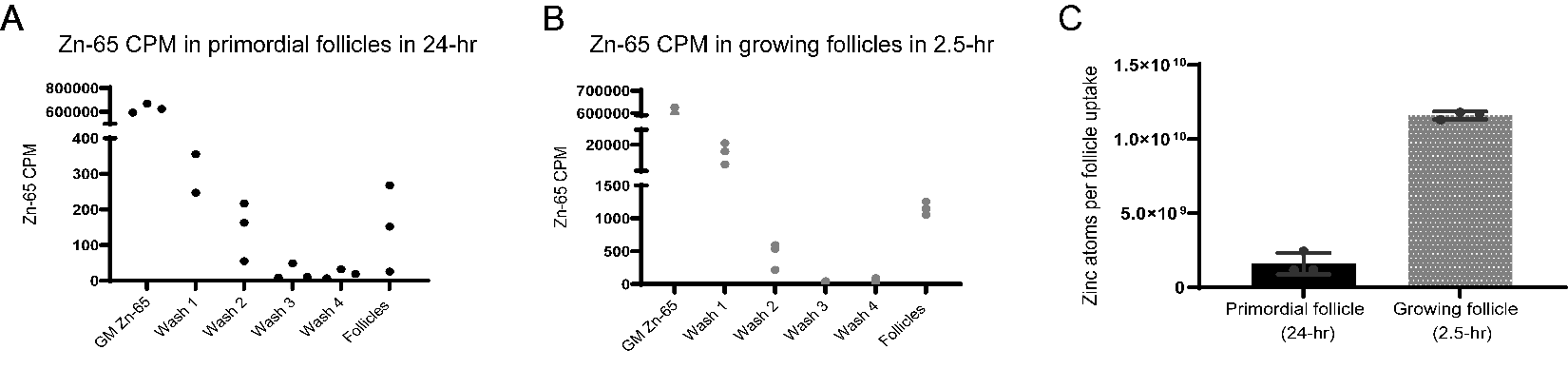


Supplementary Figure 3


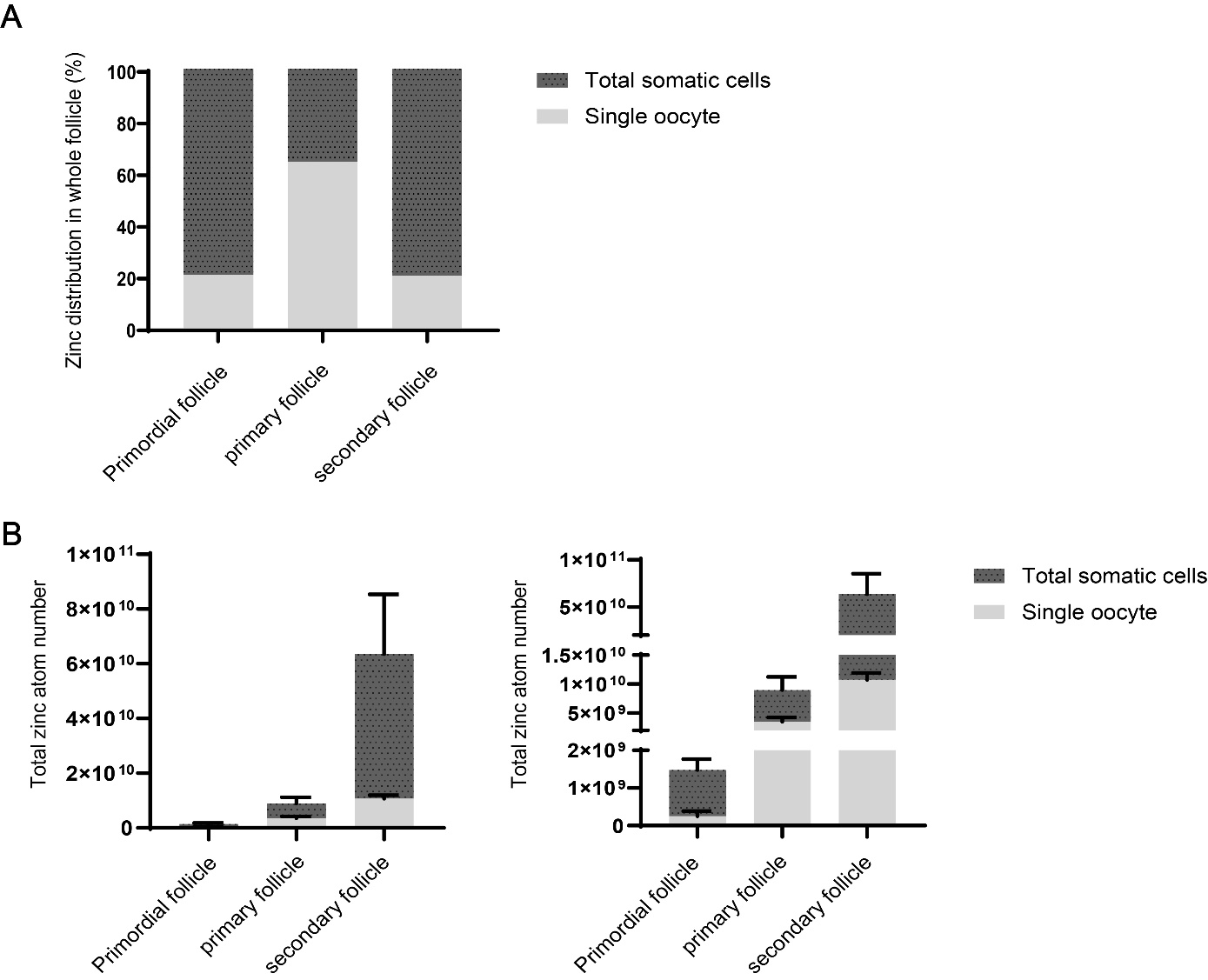


Supplementary Figure 4


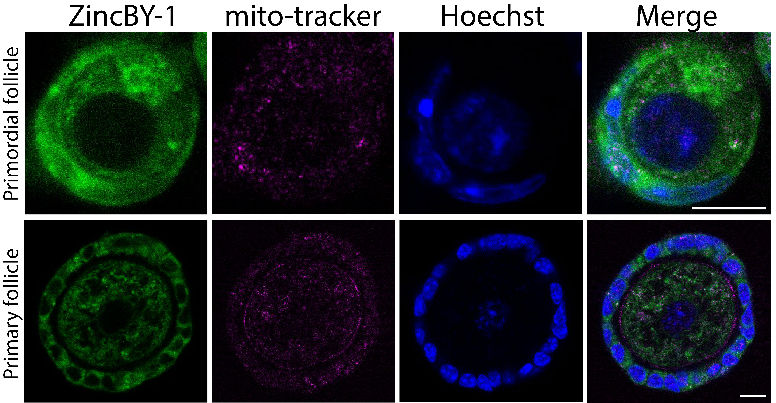


Supplementary Figure 5
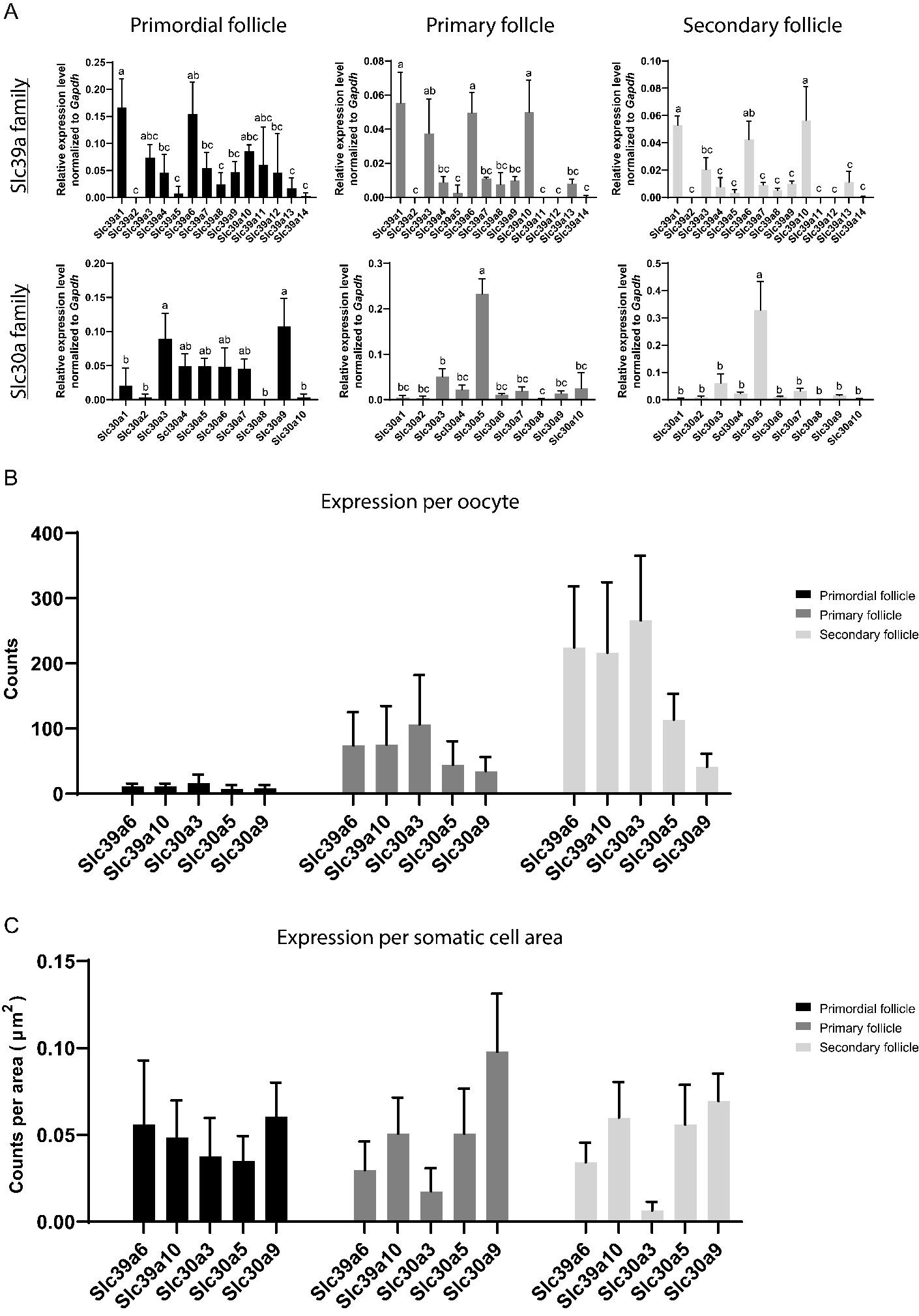


Supplementary Figure 6


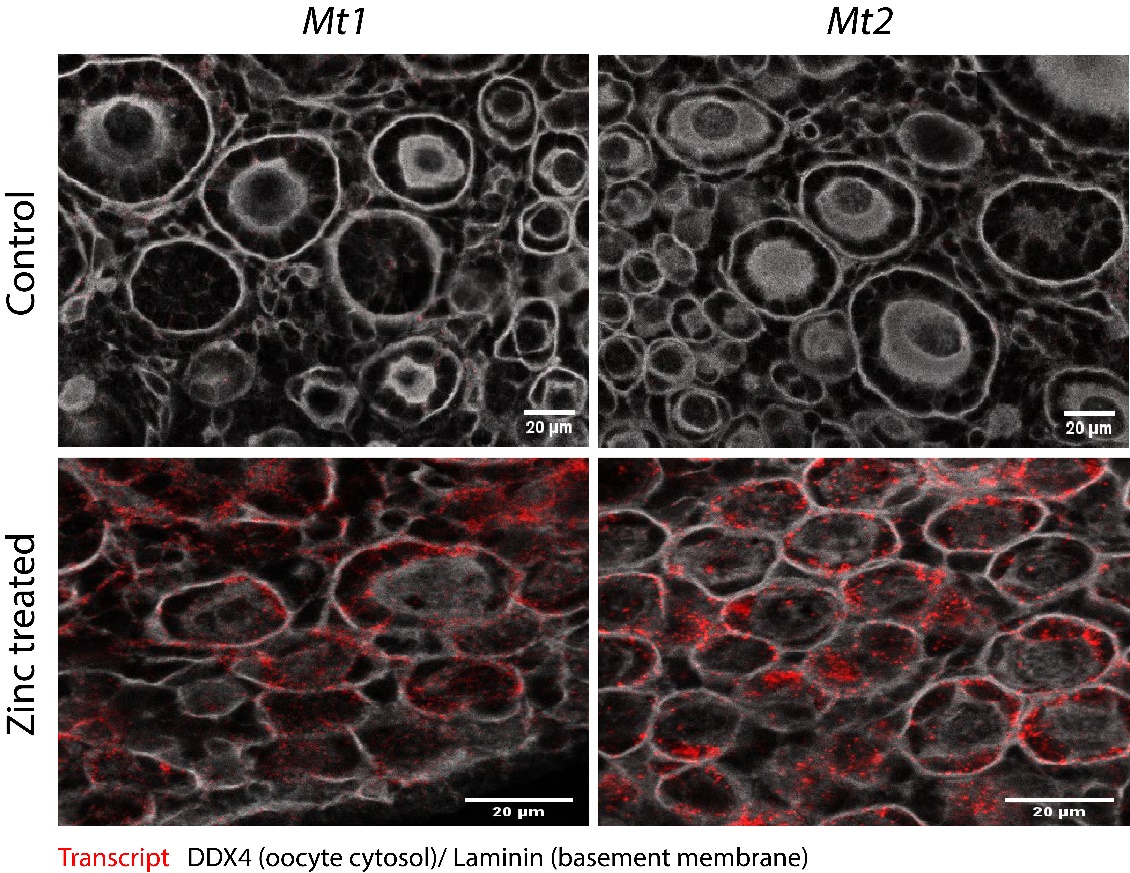


Supplementary Figure 7
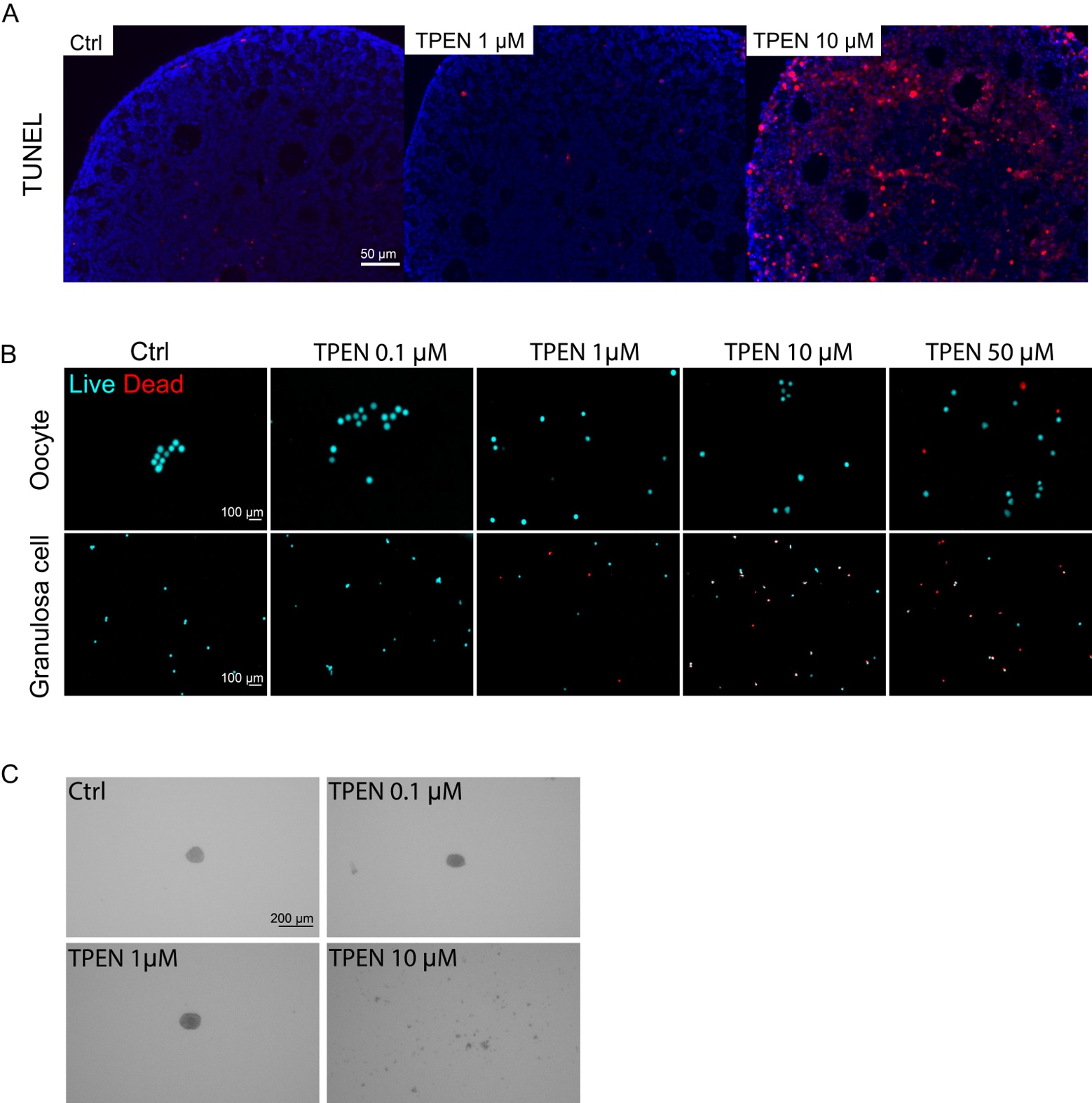


Supplementary Figure 8


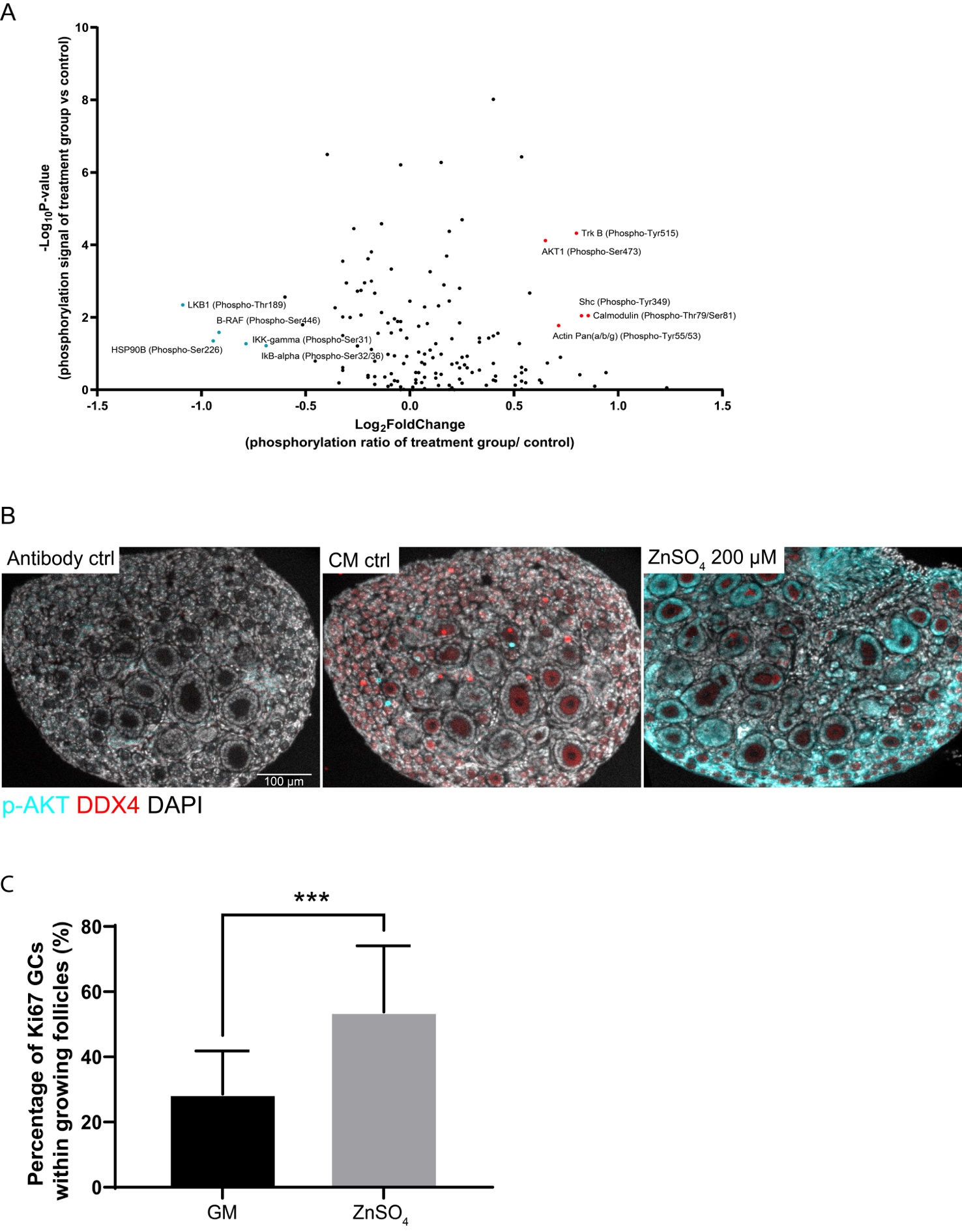


Supplementary Figure 9


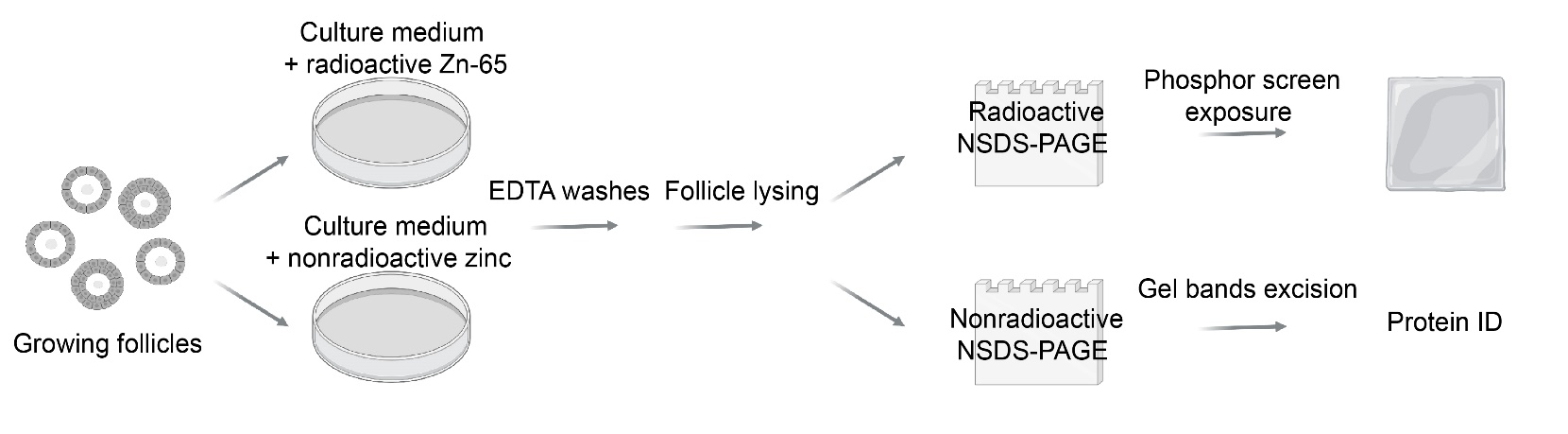


Supplementary Figure 10


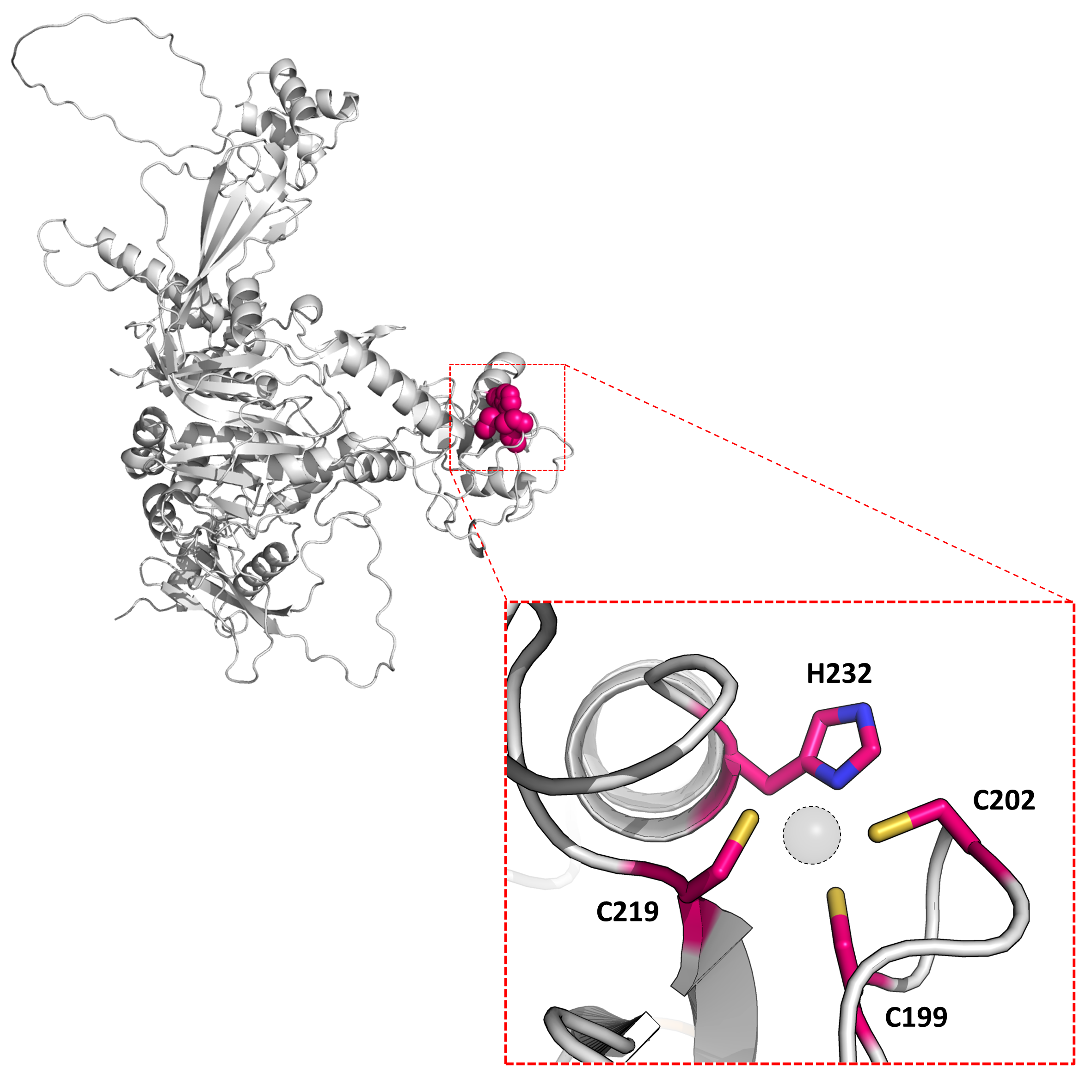


Supplementary Figure 11


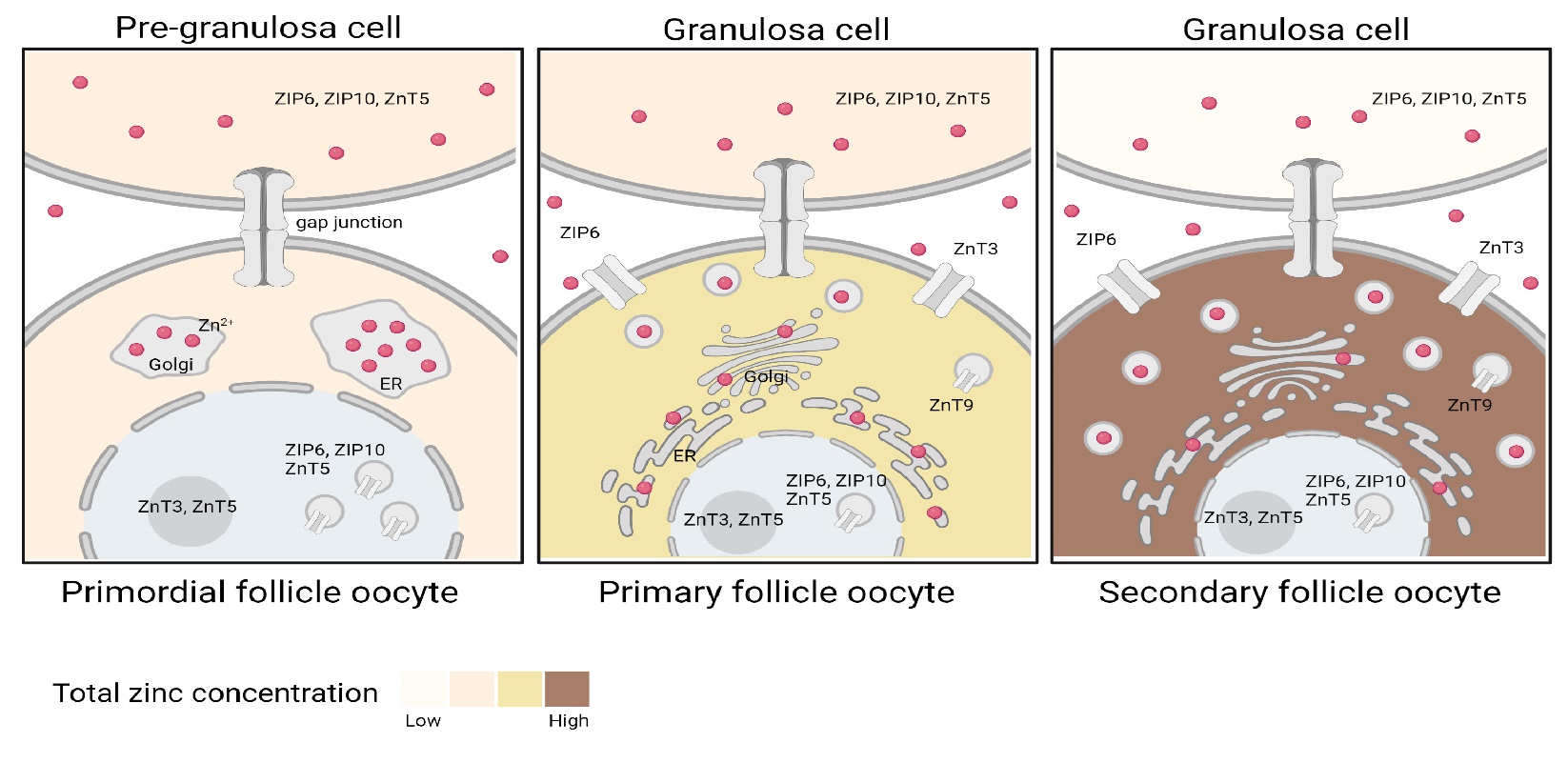


**Supplementary Tables**

Supplementary Table 1

|  | P | S | | K | | Ca | | Cr | | Mn | | Fe | | Co | | Ni | | Cu | | Zn | |
| --- | --- | --- | --- | --- | --- | --- | --- | --- | --- | --- | --- | --- | --- | --- | --- | --- | --- | --- | --- | --- | --- |
| **Primordial follicle** |  | |  | |  | |  | |  | |  | |  | |  | |  | |  | |  |
| Concentration (μg/cm^2^) | 3.75± 0.98 | | 1.46± 0.52 | | 3.48± 2.50 | | 0.03± 0.04 | | 7.01± 7.81  (10^-4^) | | 20.06± 10.36  (10^-4^) | | 150.35± 41.73  (10^-4^) | | 12.15± 3.47  (10^-4^) | | 3.67± 1.51  (10^-4^) | | 27.09± 7.69  (10^-4^) | | 259.01± 71.62  (10^-4^) |
| Total atom number | 38.58± 13.61  (10^10^) | | 14.24 ±4.94  (10^10^) | | 29.46± 23.50  (10^10^) | | 0.21± 0.22  (10^10^) | | 0.42± 0.44  (10^8^) | | 1.13± 0.56  (10^8^) | | 8.36± 2.32  (10^8^) | | 0.64± 0.17  (10^8^) | | 0.19± 0.05  (10^8^) | | 1.32± 0.31  (10^8^) | | 12.26± 2.91  (10^8^) |
| ROI area (μm^2^) | 539.38± 165.18 | | | | | | | | | | | | | | | | | | | | |
|  |  | | | | | | | | | | | | | | | | | | | | |
| **Primary follicle** |  | |  | |  | |  | |  | |  | |  | |  | |  | |  | |  |
| Concentration (μg/cm^2^) | 4.73± 0.89 | | 1.94± 0.39 | | 4.86± 2.33 | | 0.04± 0.01 | | 10.50± 3.63  (10^-4^) | | 29.60± 11.43  (10^-4^) | | 148.04± 34.42  (10^-4^) | | 14.58± 2.97  (10^-4^) | | 4.49± 0.58  (10^-4^) | | 31.65± 2.94  (10^-4^) | | 361.05± 51.31  (10^-4^) |
| Total atom number | 153.65± 71.13  (10^10^) | | 61.20± 28.92  (10^10^) | | 125.65± 89.01  (10^10^) | | 0.87± 0.36  (10^10^) | | 2.06± 1.20  (10^8^) | | 5.44± 3.43  (10^8^) | | 25.65± 10.68  (10^8^) | | 2.44± 1.06  (10^8^) | | 0.76± 0.31  (10^8^) | | 4.87± 1.71  (10^8^) | | 54.79± 22.36  (10^8^) |
| ROI area (μm^2^) | 1613.25± 498.23 | | | | | | | | | | | | | | | | | | | | |
|  |  | |  | |  | |  | |  | |  | |  | |  | |  | |  | |  |
| **Secondary follicle** |  | |  | |  | |  | |  | |  | |  | |  | |  | |  | |  |
| Concentration (μg/cm^2^) | 9.45± 4.00 | | 3.79± 1.79 | | 3.66± 2.16 | | 0.12± 0.08 | | 25.13± 16.07  (10^-4^) | | 43.79± 21.10  (10^-4^) | | 239.23± 75.91  (10^-4^) | | 26.38± 7.62  (10^-4^) | | 10.73± 3.63  (10^-4^) | | 59.83± 19.70  (10^-4^) | | 883.91± 306.44  (10^-4^) |
| Total atom number | 1190.17± 582.06  (10^10^) | | 461.50± 254.47  (10^10^) | | 369.65± 241.37  (10^10^) | | 11.69± 8.70  (10^10^) | | 18.65± 13.99  (10^8^) | | 31.15± 17.38  (10^8^) | | 168.07± 65.11  (10^8^) | | 17.48± 6.48  (10^8^) | | 7.11± 2.94  (10^8^) | | 36.60± 14.33  (10^8^) | | 528.17± 218.37  (10^8^) |
| ROI area (μm^2^) | 6374.44± 1074.49 | | | | | | | | | | | | | | | | | | | | |

Supplementary Table 2

|  | P | S | K | Ca | Cr | Mn | Fe | Co | Ni | Cu | Zn |
| --- | --- | --- | --- | --- | --- | --- | --- | --- | --- | --- | --- |
| **Primordial follicle oocyte** |  |  |  |  |  |  |  |  |  |  |  |
| Concentration (μg/cm^2^) | 1.15±0.08 | 0.59±0.03 | 0.86±0.18 | 0.03±0.00 | 3.28±1.07  (10^-4^) | 7.11±2.77  (10^-4^) | 68.74±4.66 (10^-4^) | 4.88±0.54  (10^-4^) | 1.17±0.19  (10^-4^) | 10.77±0.78  (10^-4^) | 67.58±3.40 (10^-4^) |
| Total atom number | 10.07±2.81 (10^10^) | 5.35±2.81 (10^10^) | 6.41±2.45 (10^10^) | 0.23±0.06 (10^10^) | 0.19±0.10 (10^8^) | 0.43±0.24 (10^8^) | 3.54±0.88 (10^8^) | 0.24±0.07 (10^8^) | 0.06±0.02 (10^8^) | 0.49±0.12 (10^8^) | 2.99±0.82 (10^8^) |
| ROI area (μm^2^) | 449.16±135.13 | | | | | | | | | | |
|  |  |  |  |  |  |  |  |  |  |  |  |
| **Primary follicle oocyte** |  |  |  |  |  |  |  |  |  |  |  |
| Concentration (μg/cm^2^) | 2.58±0.75 | 1.61±0.62 | 0.75±0.29 | 0.04±0.03 | 7.37±2.70  (10^-4^) | 16.67±7.26 (10^-4^) | 74.89±42.91 (10^-4^) | 7.19±3.72  (10^-4^) | 2.65±1.28  (10^-4^) | 23.27±12.23 (10^-4^) | 213.87±115.14 (10^-4^) |
| Total atom number | 96.93±23.10 (10^10^) | 56.69±11.58 (10^10^) | 25.71±14.84 (10^10^) | 1.03±0.36 (10^10^) | 1.68±0.64 (10^8^) | 3.38±0.94 (10^8^) | 14.39±5.14 (10^8^) | 1.33±0.38 (10^8^) | 0.49±0.11 (10^8^) | 3.95±0.89 (10^8^) | 35.08±7.27 (10^8^) |
| ROI area (μm^2^) | 2234.76±883.85 | | | | | | | | | | |
|  |  |  |  |  |  |  |  |  |  |  |  |
| **Secondary follicle oocyte** |  |  |  |  |  |  |  |  |  |  |  |
| Concentration (μg/cm^2^) | 5.17±0.79 | 3.27±0.56 | 2.51±0.31 | 0.03±0.02 | 16.17±8.63 (10^-4^) | 32.85±8.13 (10^-4^) | 85.17±27.38 (10^-4^) | 11.04±3.00 (10^-4^) | 5.11±1.16  (10^-4^) | 37.45±8.24 (10^-4^) | 413.90±89.09 (10^-4^) |
| Total atom number | 285.27±37.11 (10^10^) | 173.73±22.64 (10^10^) | 111.15±19.85 (10^10^) | 1.33±0.86 (10^10^) | 5.15±2.27 (10^8^) | 10.19±2.09 (10^8^) | 25.33±3.17 (10^8^) | 3.13±0.25 (10^8^) | 1.47±0.15 (10^8^) | 9.93±0.87 (10^8^) | 107.39±11.92 (10^8^) |
| ROI area (μm^2^) | 2912.88±614.84 | | | | | | | | | | |

Supplementary Table 3

|  | P | S | K | Ca | Cr | Mn | Fe | Co | Ni | Cu | Zn |
| --- | --- | --- | --- | --- | --- | --- | --- | --- | --- | --- | --- |
| **Primordial follicle somatic** |  |  |  |  |  |  |  |  |  |  |  |
| Concentration (μg/cm^2^) | 1.82±0.44 | 0.62±0.21 | 0.60±0.43 | 0.05±0.02 | 3.31±1.79  (10^-4^) | 3.97±2.38  (10^-4^) | 32.96±14.87 (10^-4^) | 3.16±1.02  (10^-4^) | 1.14±0.40  (10^-4^) | 5.74±2.22  (10^-4^) | 68.46±29.07 (10^-4^) |
| Total atom number | 2.97±1.18 (10^10^) | 0.98±0.45 (10^10^) | 0.78±0.68 (10^10^) | 0.06±0.03 (10^10^) | 3.15±1.89 (10^6^) | 3.65±2.53 (10^6^) | 31.76±22.00 (10^6^) | 2.82±1.55 (10^6^) | 0.95±0.38 (10^6^) | 4.56±2.40  (10^6^) | 53.08±29.35 (10^6^) |
| ROI area (μm^2^) | 83.39±34.30 | | | | | | | | | | |
|  |  |  |  |  |  |  |  |  |  |  |  |
| **Primary follicle somatic** |  |  |  |  |  |  |  |  |  |  |  |
| Concentration (μg/cm^2^) | 1.91±0.60 | 0.94±0.30 | 1.12±0.35 | 0.04±0.02 | 6.69±8.74  (10^-4^) | 7.76±3.08  (10^-4^) | 31.08±11.63 (10^-4^) | 3.28±1.06  (10^-4^) | 1.57±0.68  (10^-4^) | 7.61±1.78  (10^-4^) | 86.90±28.97 (10^-4^) |
| Total atom number | 2.35±0.95 (10^10^) | 1.19±0.79 (10^10^) | 1.16±0.68 (10^10^) | 0.04±0.03 (10^10^) | 6.83±4.61 (10^6^) | 5.47±2.80 (10^6^) | 22.09±10.92 (10^6^) | 2.23±1.10 (10^6^) | 1.09±0.55 (10^6^) | 4.58±1.64 (10^6^) | 52.50±24.08 (10^6^) |
| ROI area (μm^2^) | 62.78±21.98 | | | | | | | | | | |
|  |  |  |  |  |  |  |  |  |  |  |  |
| **Secondary follicle somatic** |  |  |  |  |  |  |  |  |  |  |  |
| Concentration (μg/cm^2^) | 2.08±0.40 | 1.22±0.26 | 1.54±0.56 | 0.12±0.01 | 26.62±13.00 (10^-4^) | 17.59±9.05 (10^-4^) | 39.92±20.72 (10^-4^) | 5.22±1.64  (10^-4^) | 1.83±1.02  (10^-4^) | 7.22±2.63  (10^-4^) | 54.54±17.34 (10^-4^) |
| Total atom number | 3.54±0.88 (10^10^) | 1.98±0.40 (10^10^) | 2.01±0.49 (10^10^) | 0.16±0.04 (10^10^) | 25.48±7.97 (10^6^) | 15.90±5.01 (10^6^) | 37.90±20.78 (10^6^) | 4.57±1.12 (10^6^) | 1.61.±0.98 (10^6^) | 5.95±2.25 (10^6^) | 44.24±17.51 (10^6^) |
| ROI area (μm^2^) | 89.13±19.64 | | | | | | | | | | |

Supplementary Table 4

|  | Primordial follicle stage | Primary follicle stage | GV |
| --- | --- | --- | --- |
| **ZincBY-1/ ER-tracker** |  |  |  |
| Whole follicle | 0.67 ± 0.05 (N=7) | 0.55 ± 0.07 (N=6) |  |
| Oocyte | 0.75 ± 0.10 (N=7) | 0.63 ± 0.16 (N=7) | 0.24 ± 0.12 (N=4) **** |
|  |  |  |  |
| **ZincBY-1/ Golgi-tracker** |  |  |  |
| Oocyte | 0.58 ± 0.06 (N=5) | 0.49 ± 0.06 (N=6) | 0.40 ± 0.08 (N=5) ** |
|  |  |  |  |
| **ZincBY-1/ Mitotracker** |  |  |  |
| Whole follicle | 0.33 ± 0.02 (N=4) |  |  |
| Oocyte | 0.25 ± 0.07 (N=4) |  |  |

Supplementary Table 5

| **Protein name** | **Gene name** | **Molecular weight** |
| --- | --- | --- |
| 100-200kDa group |  |  |
| DYHC1 (Cytoplasmic dynein 1 heavy chain 1 ) | Dync1h1 | 532 kDa |
| SPTN1 (Spectrin alpha chain, non-erythrocytic 1 ) | Sptan1 | 285 kDa |
| FLNA (Filamin-A ) | Flna | 281 kDa |
| FLNB (Filamin-B ) | Flnb | 278 kDa |
| SPTB2 (Spectrin beta chain, non-erythrocytic 1 ) | Sptbn1 | 274 kDa |
| TPR (Nucleoprotein TPR ) | Tpr | 274 kDa |
| MAP1B (Microtubule-associated protein 1B ) | Map1b | 270 kDa |
| TLN1 (Talin-1 ) | Tln1 | 270 kDa |
| MYH10 (Myosin-10 ) | Myh10 | 229 kDa |
| MYH9 (Myosin-9 ) | Myh9 | 226 kDa |
| EIF3A (Eukaryotic translation initiation factor 3 subunit A ) | Eif3a | 162 kDa |
| MBB1A (Myb-binding protein 1A ) | Mybbp1a | 152 kDa |
| PUR4 (Phosphoribosylformylglycinamidine synthase ) | Pfas | 145 kDa |
| CAND1 (Cullin-associated NEDD8-dissociated protein 1 ) | Cand1 | 136 kDa |
| NALP5 (NACHT, LRR and PYD domains-containing protein 5 ) | Nlrp5 | 131 kDa |
| UBA1 (Ubiquitin-like modifier-activating enzyme 1 ) | Uba1 | 118 kDa |
| MAP4 (Microtubule-associated protein 4 ) | Map4 | 117 kDa |
| NAL14 (NACHT, LRR and PYD domains-containing protein 14 ) | Nlrp14 | 113 kDa |
| KINH (Kinesin-1 heavy chain ) | Kif5b | 110 kDa |
| ACTN1 (Alpha-actinin-1 ) | Actn1 | 103 kDa |
| PYGB (Glycogen phosphorylase, brain form ) | Pygb | 97 kDa |
| ***UBP5 (Ubiquitin carboxyl-terminal hydrolase 5 )*** | ***Usp5*** | ***96 kDa*** |
| HSP74 (Heat shock 70 kDa protein 4 ) | Hspa4 | 94 kDa |
| PLAK (Junction plakoglobin ) | Jup | 82 kDa |
| TLE6 (Transducin-like enhancer protein 6 ) | Tle6 | 65 kDa |
| HYES (Bifunctional epoxide hydrolase 2 ) | Ephx2 | 63 kDa |
| DPYL2 (Dihydropyrimidinase-related protein 2 ) | Dpysl2 | 62 kDa |
| DPYL3 (Dihydropyrimidinase-related protein 3 ) | Dpysl3 | 62 kDa |
| TCPB (T-complex protein 1 subunit beta ) | Cct2 | 57 kDa |
| CAP1 (Adenylyl cyclase-associated protein 1 ) | Cap1 | 52 kDa |
| GDIB (Rab GDP dissociation inhibitor beta ) | Gdi2 | 51 kDa |
| NPM (Nucleophosmin ) | Npm1 | 33 kDa |
| Dye front group |  |  |
| ANXA2 (Annexin A2) | Anxa2 | 76 kDa |
| RS6 (40S ribosomal protein S6) | Rsp6 | 29 kDa |

Supplementary Table 6

|  | *Mouse UBP5* |  |
| --- | --- | --- |
| Type of Zinc site | Residues | Precision Score |
| C3H1 | CYS199, CYS202, CYS219, HIS232 | 1.00 |
| D1H1 | ASP435, HIS809 | 1.00 |
| D1H1 | ASP119, HIS72 | 1.00 |
| D1H1 | ASP269, HIS179 | 1.00 |
| D1H1 | ASP214, HIS236 | 1.00 |
| D1H1 | ASP262, HIS179 | 1.00 |
| E1H2 | GLU427, HIS425, HIS442 | 1.00 |
| E1H1 | GLU388, HIS382 | 0.96 |
| E1H1 | GLU263, HIS179 | 0.96 |

Supplementary Table 7

| **Primary Antibody** | **Vendor** | **Catalog Number** | **Dilution** |
| --- | --- | --- | --- |
| P-AKT | Invitrogen | 44-621G | 1:100 |
| DDX4 | abcam | ab27591 | 1:100 |
| Ki-67 | Invitrogen | 14-5698-82 | 1:100 |
| ZIP6 | Homemade (Genscript 0.586 mg/mL) | NA | 1:50 |
| ZIP10 | Homemade (Prosci, 5 mg/mL) | NA | 1:1000 |
| ZnT3 | Bioss | bs-8717R | 1:50 |
| ZnT5 | Homemade (2.5 mg/mL) | NA | 1:500 |
| ZnT9 | My Bioscource | MBS9606146 | 1:100 |
| Fibrillarin | abcam | ab4566 | 1:100 |
| Laminin | Novus | NB300-144 | 1:100 |
| UBP-5 | Invitrogen | PA5-52341 | 1:100 |
|  |  |  |  |
| **Secondary Antibody** | **Vendor** | **Catalog Number** | **Dilution** |
| Donkey anti- rabbit AF488 | Invitrogen | A32790 | 1:200 |
| Donkey anti- mouse AF568 | abcam | 175700 | 1:200 |
| Goat anti- rat AF488 | Invitrogen | A11006 | 1:200 |
|  |  |  |  |

Supplementary Table 8

| **Primer** | **Sequence (5' - 3')** | **Primer** | **Sequence (5' - 3')** |
| --- | --- | --- | --- |
| Slc39a1-F | GGT CTC TCT GCC AGT TTT CG | Slc30a1-F | GCT CTC GAG TTG GTC CTG TC |
| Slc39a1-R | CAG CAT TAA GGA GGC AGA GG | Slc30a1-R | GCC TCA TGG TGA GGT AGG AA |
| Slc39a2-F | CCT GCT TGC TCT TCT GGT TC | Slc30a2-F | TTC TGG AAG TCA CCC TGA CC |
| Slc39a2-R | CCT CCA GAG CTT CAG CAG TC | Slc30a2-R | CTA ATG AGC ATG CTG GCA AA |
| Slc39a3-F | CCA TGG TTC ACA CAC AGA GG | Slc30a3-F | CCA TCA GCA CCT TCC TCT TC |
| Slc39a3-R | AGG GTC CCT GAG GTC ACT TT | Slc30a3-R | ATG GAG ATC ATG GGT TGC TC |
| Slc39a4-F | CTT GGC TCT AGG CAA ACC TG | Slc30a4-F | TGC CGT CCT CTA CTT GCT TT |
| Slc39a4-R | AGT GTG GCC AGG TAA TCG TC | Slc30a4-R | TAG GCG ATG AAA TCC AAA GG |
| Slc39a5-F | GCC AGA GGG AGA ACA GAC AG | Slc30a5-F | TTG GTT TTC ATA CGG CTT CC |
| Slc39a5-R | GTG GCA GAA GAC TGC TAG GG | Slc30a5-R | TTT GGA CAC GTC CAT TTT GA |
| Slc39a6-F | TTC CTG TCT CTG CTG GGA GT | Slc30a6-F | GTC CAC GCT GAC TGT TCA GA |
| Slc39a6-R | TGT GCT GAT GAC TTG CAT GA | Slc30a6-R | CGT TTT TCC CAG GCG TAT TA |
| Slc39a7-F | AGG AGT GTC AGC CTT GGA GA | Slc30a7-F | GCC ACC ATA CCG AGT CAC TT |
| Slc39a7-R | ATT AGG GAC CAT CGG GTA GG | Slc30a7-R | CCA CAA AAG CGA AAG AGA GG |
| Slc39a8-F | AGC CTA ACG GAC ACA TCC AC | Slc30a8-F | ACT GAT GCG GCT CAT CTC TT |
| Slc39a8-R | AGT ACA AGA TGC CCC AAT CG | Slc30a8-R | GAT GCA AAG GAC AGA CAG CA |
| Slc39a9-F | GCA TTA GAG GCA GCA GGA AC | Slc30a9-F | GTC ACC CAC GGT CTC TCA TT |
| Slc39a9-R | GCA TTA AGG CAT CCA CAC CT | Slc30a9-R | ACT GCC TGT TCC AAA ACC AC |
| Slc39a10-F | TAC CCA CCA GCA TTT TCA CA | Slc30a10-F | TGT GCA TGC TAA GGA ACT GC |
| Slc39a10-R | TCA CTG TGA GCA ACG GAG TC | Slc30a10-R | CAC GGT CCA AGA ATG GAC TT |
| Slc39a11-F | CTT CTT CAC CTG GGC AAT GT |  |  |
| Slc39a11-R | GGA GGT CAG CCA GGT AGA CA | GAPDH-F | GCC GAG AAT GGG AAG CTT GTC AT |
| Slc39a12-F | GAC TGC AAG CTG TGT TTG GA | GAPDH-R | GTG GTT CAC ACC CAT CAC AAA CAT |
| Slc39a12-R | CTA AGG CCG AGT AGG CTG TG |  |  |
| Slc39a13-F | GCC TGT CGC CTG GAT AAT AA |  |  |
| Slc39a13-R | CCA CCT AAG GCA AAG CTG AG |  |  |
| Slc39a14-F | TCA GCC GTG TGC TCA CTT AC |  |  |
| Slc39a14-R | GGT GCT CGT TTT TCT GCT TC |  |  |

Supplementary Table 9

| **Probe** | **Catalog Number** |
| --- | --- |
| Mm-Slc39a1 | 572121 |
| Mm-Slc39a6 | 569401 |
| Mm-Slc39a10-C2 | 588101-C2 |
| Mm-Slc30a3-C3 | 496291-C3 |
| Mm-Slc30a5-C2 | 588111-C2 |
| Ms-Slc30a9-C3 | 564731-C3 |
| Mm-Mt1-C3 | 547711-C3 |
| Mm-Mt2-C2 | 588111-C2 |
